# Supplementary figures and images for: A dose-response model for statistical analysis of chemical genetic interactions in CRISPRi screens
Source: PLoS Comput Biol. 2024 May 20;20(5):e1011408. doi: 10.1371/journal.pcbi.1011408 (PMC11104602; doi:10.1371/journal.pcbi.1011408)

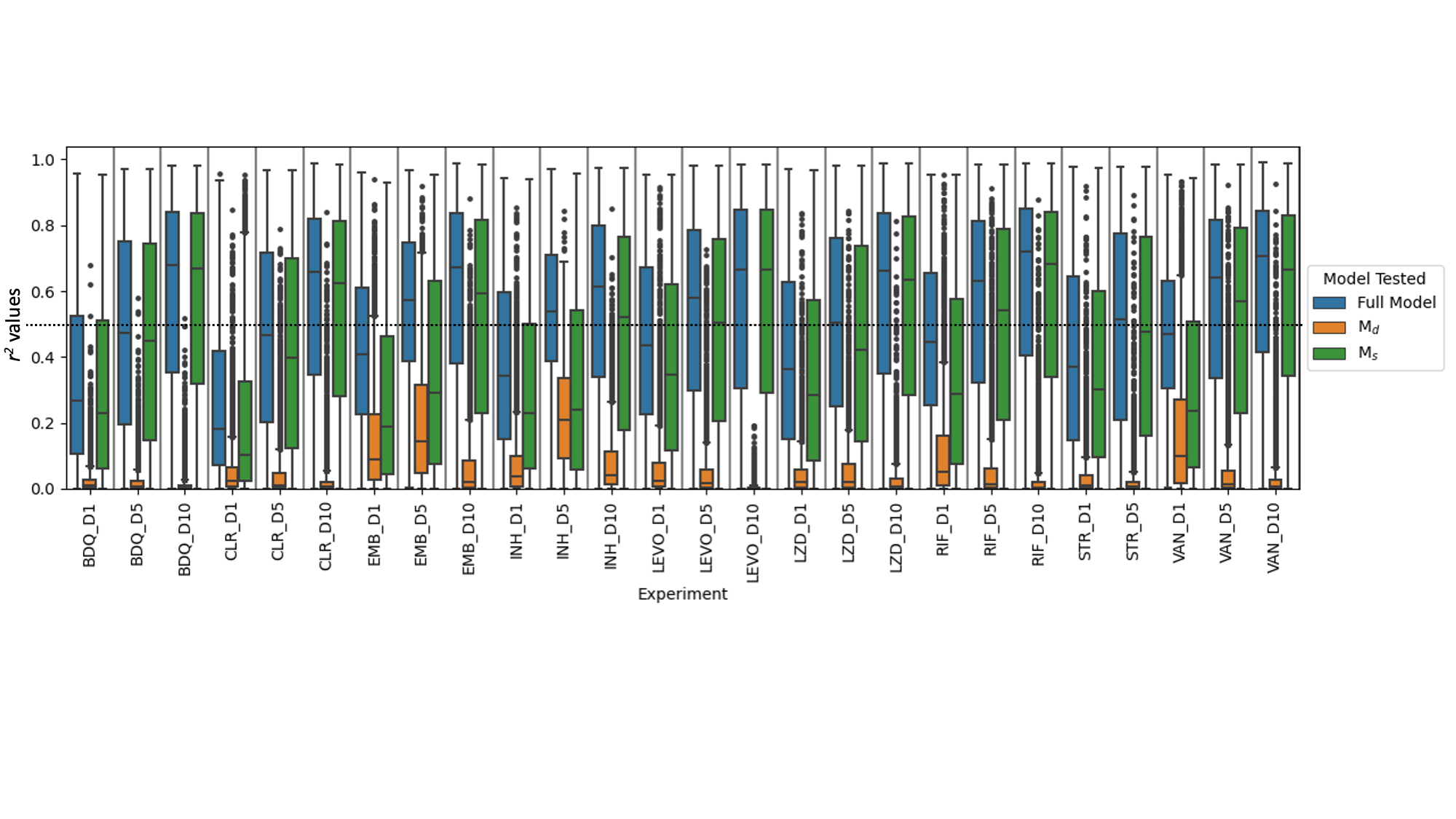

Supplement: S1 Fig — The horizontal line is where r2 = 0.5. The average r2 Ms model for all genes across all the experiments is 0.42, the average r2 for the Md model is 0.07. This, along with the AIC comparisons and Log-likelihood tests, indicate sgRNA efficiency is the more significant predictor. However, the full CRISPRi-DR model outperforms both Md and Ms (average r2 is 0.50) indicating the inclusion of both sgRNA efficiency and log concentration is needed for accurate assessment of significant sgRNA depletion in a gene in a condition. (TIFF) [file pcbi.1011408.s001.tiff]

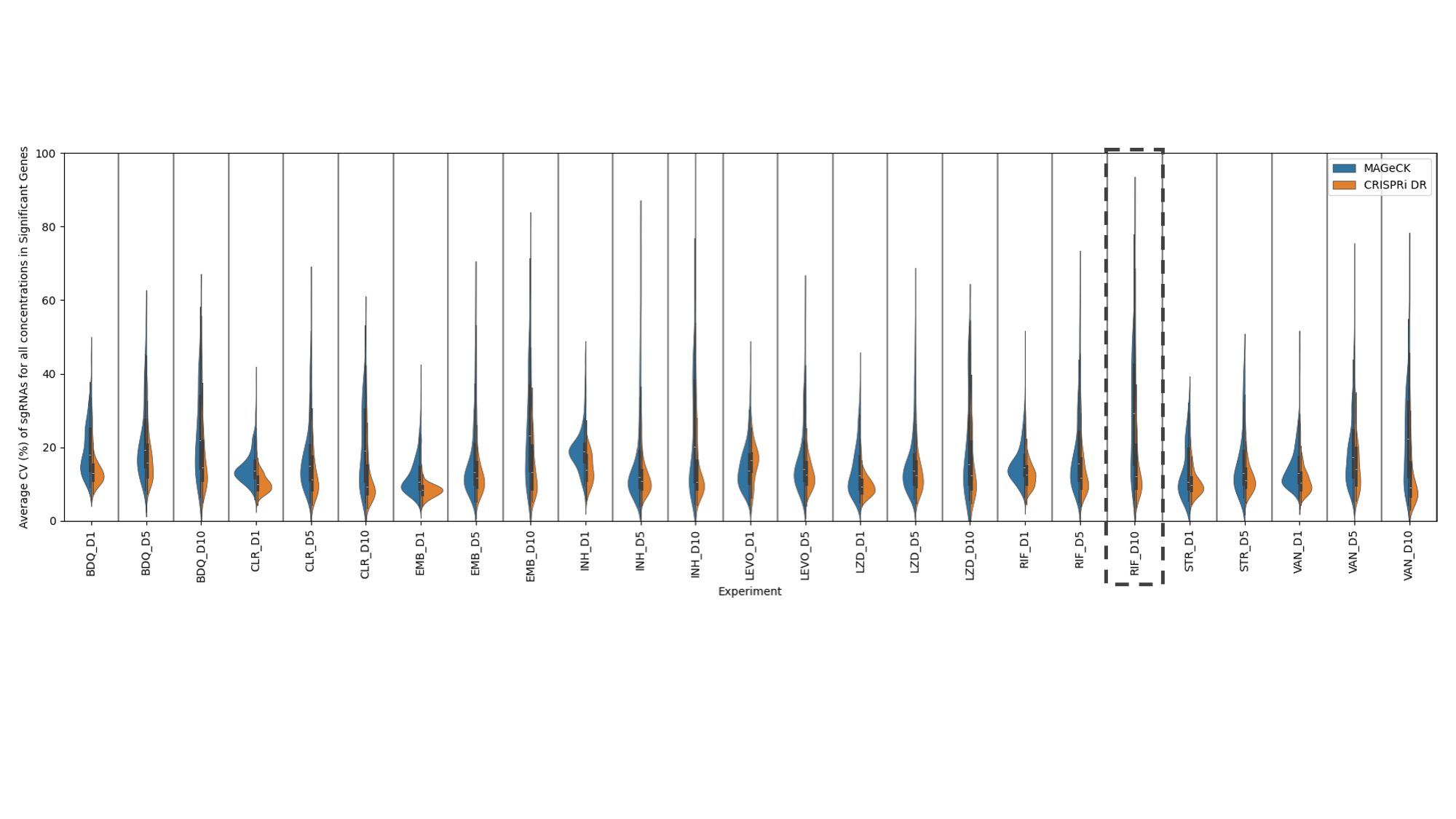

Supplement: S2 Fig — Comparisons of distributions of coefficient of variation (noise) for significant genes found by MAGeCK and the CRISPRi-DR model for all experiments. The dashed panel is the noise distributions for RIF D10, seen in Fig 5. The trend seen in RIF D10 is present with all the experiments except LEVO D10. The distribution of noise for hits found by the CRISPRi-DR model is unimodal with a low CV as the mode, whereas MAGeCK finds significant genes with low average CV values but also a substantial amount of genes with high average CV values. LEVO D10 was left out of this plot due to the low number of hits in either model. (TIFF) [file pcbi.1011408.s002.tiff]
