# Supplementary material for: A dose-response model for statistical analysis of chemical genetic interactions in CRISPRi screens
Source: PLoS Comput Biol. 2024 May 20;20(5):e1011408. doi: 10.1371/journal.pcbi.1011408 (PMC11104602; doi:10.1371/journal.pcbi.1011408)
Supplement: S1 Text — We expand on the following four topics from the main text in this document: 1) An assessment of CRISPRi-DR, MAGeCK and MAGeCK-MLE on datasets with simulated noise, 2) Comparison of CRISPRi-DR to other analysis methods using CGI datasets, 3) Analysis of E. coli CRISPRi screens using CRISPRi-DR and, 4) The minimum number of sgRNAs recommended per gene in CRISPRi-DR. (PDF) [file pcbi.1011408.s006.pdf]

A dose-response based model for statistical analysis of chemical genetic interactions in CRISPRi screens

Sanjeevani Choudhery, Michael A. DeJesus, Aarthi Srinivasan, Jeremy Rock, Dirk Schnappinger, Thomas R. Ioerger

Supplementary Material

We expand on the following four topics from the main text in this document:

1. An assessment of CRISPRi-DR, MAGeCK and MAGeCK-MLE on datasets with simulated noise (p. 1)
2. Comparison of CRISPRi-DR to other analysis methods using CGI datasets (p. 15)
3. Analysis of *E. coli* CRISPRi screens using CRISPRi-DR (p. 25)
4. The minimum number of sgRNAs recommended per gene in CRISPRi-DR. (p. 29)

---

1. Assessment of CRISPRi-DR, MAGeCK and MAGeCK-MLE on datasets with simulated noise

Simulation Design

To better understand the differences in performance, between CRISPRi-DR, MAGeCK-RRA, and MAGeCK-MLE, and asses their sensitivity to various sources of noise, we developed a hierarchical simulation model.

In this experiment, we simulated 1000 genes with 20 sgRNAs each. The first 50 genes are chosen as true negative interaction (with a virtual drug), the second 50 as positive interactions, and the last 50 as negative controls (for MAGeCK-RRA and MAGeCK-MLE). We simulated exposure to a virtual inhibitor over 4 concentrations (1μM, 2μM, 4μM, and 8μM), 3 replicates each. Our objective was to quantify how much noise in the counts, both within concentrations and between concentrations, affects the precision and recall of each method.

We sample parameters for baseline abundances, sgRNA efficiencies, and drug sensitivities (concentration-dependence) from prior distributions. Then, we use the dose-response model (sigmoid transformation of linear model) to generate mean counts for each sgRNA at each concentration (see Eq. 1 below). Finally, we draw multiple replicates of actual counts surrounding these means from a Negative Binomial distribution while creating variance/noise in the observations (Eq. 2 below).

<u>Sampling Baseline Counts</u>

Baseline barcode counts were generated for each sgRNA *j* by sampling from a LogNormal distribution:

$$B_j \sim LogNormal(shape = exp(5), scale = 1)$$

This produces baseline counts in the range of single digits up to a few thousand, with a mean count in the hundreds (see histogram), which is typical of what is seen in real CRISPRi sequencing datasets.

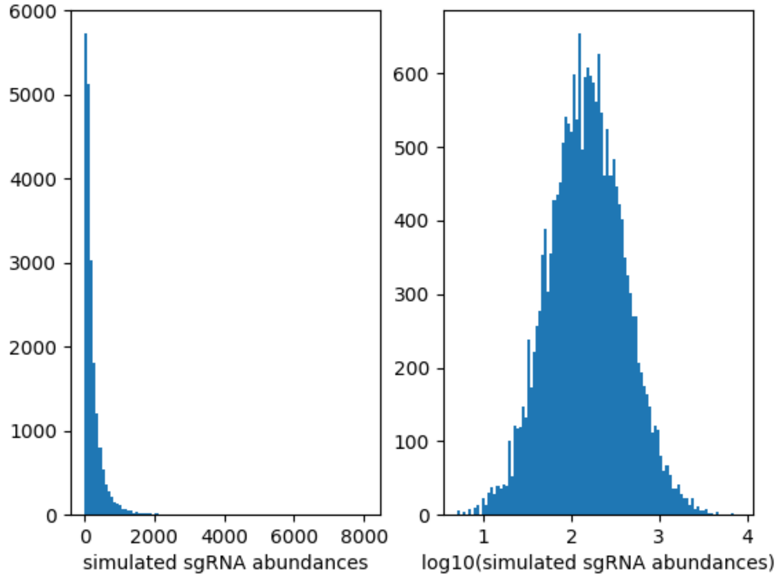

Sampling sgRNA efficiencies

To simulate the effect of CRISPRi depletion, an efficiency is chosen for each individual sgRNA j in each gene i from a uniform distribution:

$$S_{ij} \sim U(-25,0)$$

This range was chosen based on the data from the *Mtb* CRISPRi library in the Li, Poulton (1) paper, which was based on empirical estimates of growth rates (fitness defects), fit to a piecewise linear model and extrapolated to predicted LFCs at 25 generations [2]. Since sgRNA efficiencies empirically span a range of -25 to 0, and we want 0 to represent no depletion and -25 to represent high depletion (in induced vs. uninduced).

Sampling concentration-dependent slopes

Next, concentration-dependent coefficients (slopes) are chosen for each gene i. For interacting genes, slopes are chosen from a Normal distribution around $+K_2$ or $-K_2$, where K_2 is a parameter. For non-interacting genes, slopes are chosen from a Normal distribution around 0, with a standard deviation of σ_{K2}. The larger the variance, the more risk there is the slopes of non-interacting genes overlapping with interacting genes.

$$M_i \sim \left\{ \begin{matrix} \mathrm{N}(0, \sigma_{K2}^2) \text{ if } i \text{ is a non} - \text{interacting gene} \\ \mathrm{N}(+\mathrm{K}_2, \sigma_{K2}^2) \text{ if } i \text{ is a positive interaction} \\ \mathrm{N}(-\mathrm{K}_2, \sigma_{K2}^2) \text{ if } i \text{ is a negative interaction} \end{matrix} \right\}$$

Using K_2=0.8 and σ_{K2}=0.2, the histogram below shows the overlapping distributions of slopes for the 3 types of genes.

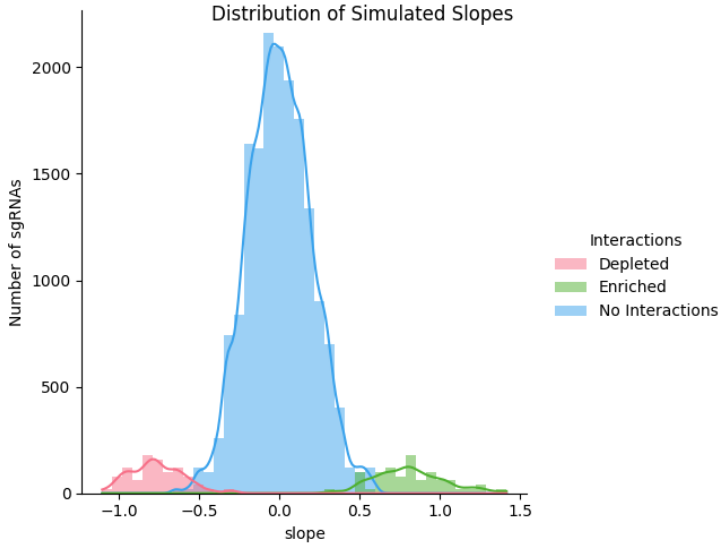

Simulation of sgRNA counts

Next, mean abundances for each sgRNA at each concentration were generated by sampling from the dose-response equation. First, a linear model was used to estimate a level which was then transformed to an abundance (A_{ijc}) by the sigmoid transformation, σ, reproducing the modified dose-response equation described in the main text.

$$A_{ijc} \ = \ B_j * \sigma(\, K_0 + K_1 * S_{ij} + M_i * log(\frac{c}{q}) \,) \qquad (1)$$

$$\sigma(x) = \frac{exp(x)}{1 + exp(x)}$$

Four concentrations were simulated: 1, 2, 4, 8 times the IC_{50} (q). Thus, log-concentrations used in the linear model were 0, 1, 2, 3. The IC_{50} is represented by q; a constant IC_{50} of q=2.0 was used.

The coefficients K_0 and K_1 were set to 3 and 0.3, respectively, to simulate sensitivity to protein depletion.

Finally, given the level of abundance for each sgRNA at each concentration, 3 replicates (x) were drawn from a Negative Binomial distribution. The variability (noisiness) of the raw counts could be adjusted by manipulating the probability parameter p=P_{nb}, which was combined with the target mean abundance (A_{ijc}) to determine the size parameter $r = A_{ijc} * \frac{Pnb}{1-Pnb}$ for the sampling:

$$Y_{ijcx} \sim NegativeBinomial(p, r) \qquad (2)$$

$$p = Pnb \, ; \, r \ = \ A_{ijc} * \frac{p}{1-p}$$

Simulating noise between concentrations

In addition to noise between replicate counts (controlled by P_{nb}) and noise affecting drug sensitivity (variability of slopes, controlled σ_{K2}), another source of noise was simulated by randomly shifting the abundance at each concentration, where the shifts were sampled from a Normal distribution, inflating or deflating the expected counts at a given concentration, deviating from a perfect linear trend. This was modeled as a gene-level effect using the parameter σ_C controls the noise between concentrations.

$$D_{ic} \sim N(0, \sigma_C)$$
$$A_{ijc}' = exp(D_{ic}) * A_{ijc}$$

Results

The other parameters are set as the following:

- sgRNA efficiency sensitivity: K_0=3, K_1=0.3
- concentration dependence: q=2, K_2=0.8, σ_{K2}=0.2

We generated simulated datasets by varying the 2 noise parameters:

- noise between concentrations: low: σ_C =0.01, med: σ_C =0.08 ,high: σ_C =0.16
- noise between replicates: low: P_{nb}=0.9, med: P_{nb}=0.5, high: P_{nb}=0.1

By forming all combinations of these parameters, we generated the following 9 scenarios:

- LL (Low noise between concentrations, Low noise within concentrations): σ_C =0.01, P_{nb}=0.9
- LM (Low noise between concentrations, Medium noise within concentrations): σ_C =0.01, P_{nb}=0.5
- LH (Low noise between concentrations, High noise within concentrations): σ_C =0.01, P_{nb}=0.1
- ML (Medium noise between concentrations, Low noise within concentrations): σ_C =0.08, P_{nb}=0.9
- MM (Medium noise between concentrations, Medium noise within concentrations): σ_C =0.08, P_{nb}=0.5
- MH (Medium noise between concentrations, High noise within concentrations): σ_C =0.08, P_{nb}=0.1
- HL (High noise between concentrations, Low noise within concentrations): σ_C =0.16, P_{nb}=0.9
- HM (High noise between concentrations, Medium noise within concentrations): σ_C =0.16, P_{nb}=0.5
- HH (High noise between concentrations, High noise within concentrations): σ_C =0.16, P_{nb}=0.1

Fig 1 illustrates the different effects of simulated noise between concentrations vs. within concentrations (among replicates) in the low-high combination scenarios, for representative sgRNAs in negative interacting genes. The decreasing trend of counts is more variable in HL and HH than in the LL and LH scenarios. Whereas the dispersion of counts within a concentration is greater in LH and HH. The medium noise scenarios (LM, ML, MM, MH, HM) are somewhere in between these four levels of dispersions.

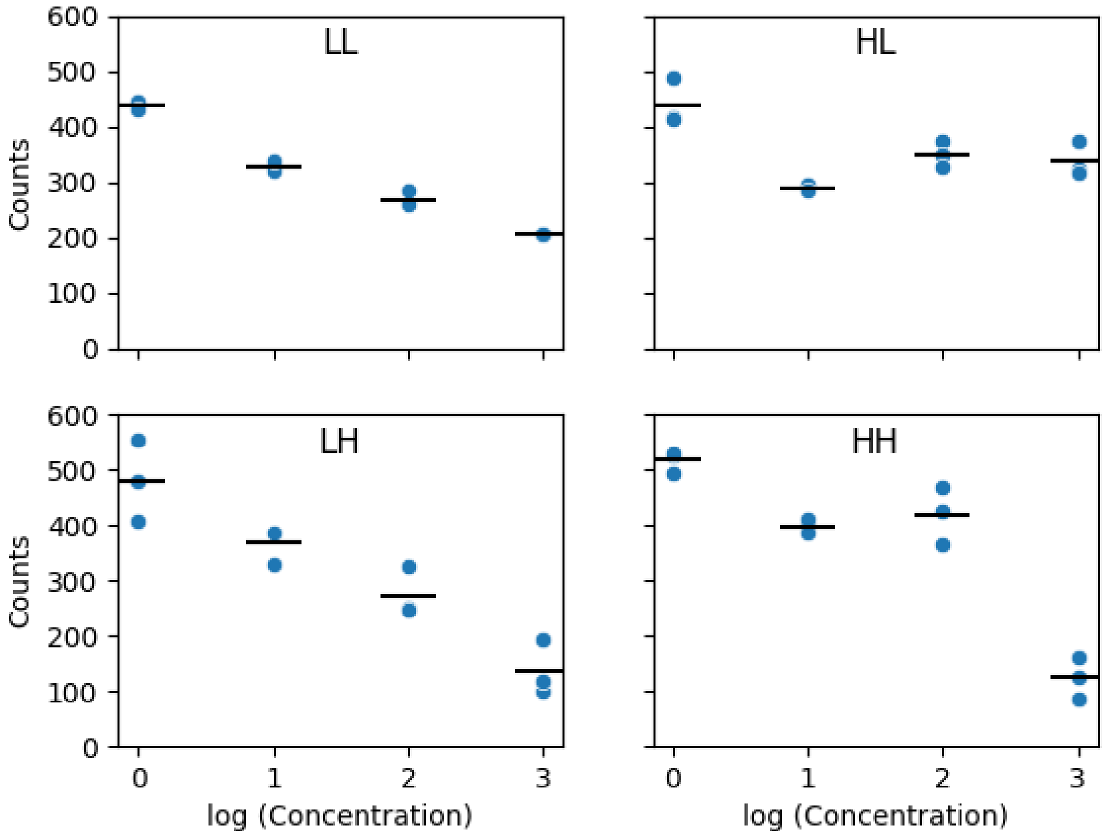

Fig 1. Abundances of select sgRNA(s) in the low and high noise scenarios. For each noise scenario, the counts of a representative sgRNA from simulated negative interacting genes are seen here with the means across replicates marked by the horizontal black line. The medium noise scenarios are somewhere in between levels of dispersion depicted.

We analyzed all 9 scenarios with MAGeCK-MLE, MAGeCK-RRA and CRISPRi-DR. For CRISPRi-DR, the criteria used to identify significant interactions was adjusted P-value<0.05 and empirical Bayes FDR <0.05, as described in the main text. For MAGeCK-MLE, the criteria used to identify significant interactions was adjusted P-value based on Wald < 0.05. MAGeCK was run 3 times independently for each drug concentration: 2 μM, 4 μM, 8 μM. Each was compared to the simulated no-drug control (DMSO). A single P-value per gene was calculated from the three analyses using the Fisher's method, which was then adjusted using Benjamini-Hochberg. Additionally, a single LFC value per gene was set equal to the most significant LFC across the three concentrations. Significant interactions were classified as combined adjusted P-value < 0.05 and |gene LFC| >1.

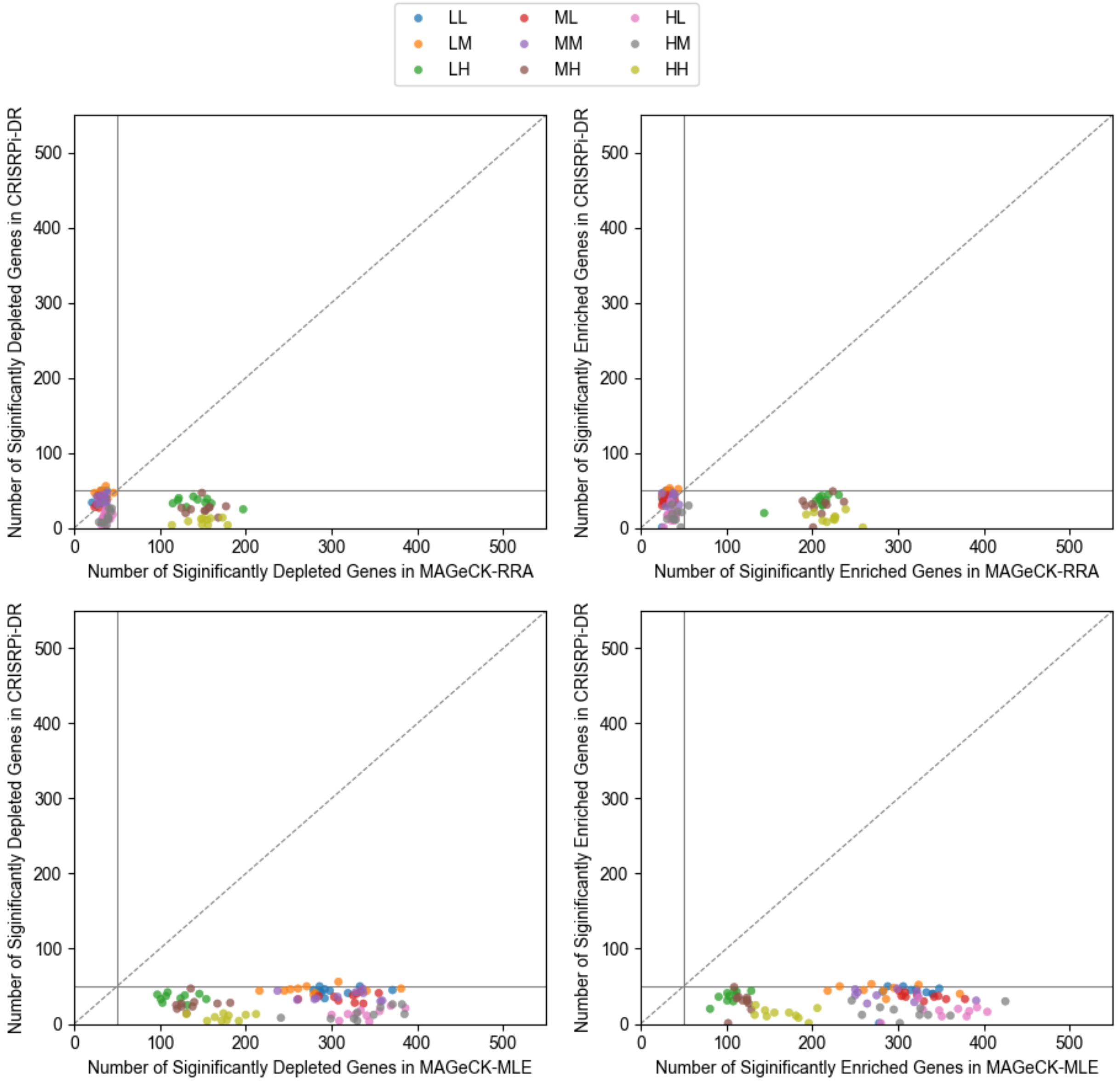

Fig 2. Comparisons of significant number of depleted and enriched genes reported by the CRISPRi-DR, MAGeCK and MAGeCK-MLE. The top two panels are comparison of CRIPSRi-DR to MAGeCK and bottom panels are comparison to MAGeCK-MLE. The left panels are comparisons of depleted genes, and the right panels are comparisons of enriched genes. The number of hits (both enriched and depleted) are greater in MAGeCK-MLE than in the CRISPRi-DR model and in some cases MAGeCK-RRA, with the greatest difference coming from HH noise datasets.

The top two panels of Fig 2 show the number of significant hits detected by CRISPRi-DR and MAGeCK-RRA, mirroring Fig 4 in the main text. The solid lines represent the 50 true positives (simulated interacting depleted or enriched genes). The dashed diagonal line is a y=x line. Although, CRISPRi-DR reports a number of genes within the same order of magnitude as MAGeCK-RRA, consistent with observations made with the experimental data, CRISPRi-DR's reported significant gene count spans a shorter range than MAGeCK-RRA's. Note that the number of significant genes reported by MAGeCK-RRA by adjust P-

value significance was further restricted with a LFC cut-off (|LFC|>1). Without this LFC filter, the number of significant genes by MAGeCK-RRA is too large to be meaningfully compared. We can see that the datasets with the highest noise have the largest discrepancies between the number of hits for both depleted and enriched genes. Thus, we can speculate that the experimental datasets seen in Fig 5 with higher discrepancy are those with high noise resulting in a possibly higher number of false positives. Comparatively as seen in the bottom two panels of Fig 2, the discrepancies between CRISPRi-DR and MAGeCK-MLE are high for all datasets, regardless of noise. CRISPRi-DR hits do not exceed the number of simulated interacting genes for both depleted and enriched cases, whereas MAGeCK-MLE nearly always does. Given there were 50 simulated depleted/enriched genes, the up to 300 depleted or enriched calls made by MAGeCK-MLE include many false positives.

Table 1 shows detailed confusion matrices of the calls made by the three methods, using the 50 simulated negative and 50 simulated positive interactions (genes 0-49 and genes 50-99) as the ground truth. Correct predictions are on the descending diagonals, and the off-diagonal entries represent errors (either false positives, FP, or false negatives, FN). The center of these matrices are correctly identified non-interacting genes. This is the largest square in all noise scenarios, for all three methods and is excluded from recall/precision calculations, to focus on predictive performances for depleted and enriched genes.

MAGeCK-MLE identifies a large number of interacting genes for all the simulated datasets and as a result has a consistently high recall rate. For example, in the lowest noise scenario (LL), CRISPRi-DR identified 79.8% of the simulated interacting genes, MAGeCK-RRA identifies 56.2% and MAGeCK-MLE identifies 99.9% of the genes. However, this also means MAGeCK-MLE makes many calls that are false positives. CRISPRi-DR falsely identifies an average of 4.6 genes, MAGeCK false identifies 3.9 genes and MAGeCK-MLE identified 519.7.

As noise is increased through adjustments of the σ_C or P_{nb} parameters, the number calls made by MAGeCK increase, thus increasing its recall rate to be similar to that of MAGeCK MLE. In the HH scenario, the recall rate of MAGeCK-MLE remains quite high at 96.8% and MAGeCK-RRA's increases to 86.3%. Conversely, with the increased noise, CRISPRi-DR makes fewer calls resulting in a decreased recall rate of 21.0% in the HH scenario. However, CRISPRi-DR sustains a low false positive rate at 0.2%. Whereas the false positive rates of MAGeCK-MLE and MAGeCK increase substantially (MLE = 27.5%, RRA = 33.7%), diluting the sets of predicted enriched and depleted genes with non-interacting genes. In summary, with increasing noise CRISPRi-DR identifies less of the true interacting genes, yet maintains its ability to keep the set of reported interacting genes from being diluted with non-interacting genes.

Table 1. Evaluation of CRISPRi-DR, MAGeCK-RRA, and MAGeCK-MLE performances in the nine noise scenarios. For each of the noise scenarios, CRISPRi-DR, MAGeCK-RRA and MAGeCK-MLE are run. These confusion matrices reflect the average confusion matrix of the 10 runs per noise scenario.

scenario LL			CRISPRi-DR predictions			MAGeCK-RRA predictions			MAGeCK-MLE predictions		
			Depleted	Non-Interacting	Enriched	Depleted	Non-Interacting	Enriched	Depleted	Non-Interacting	Enriched
	Simulated Gene Types	Depleted	41.2	8.8	0	29.5	20.5	0	49.9	0.1	0
		Non-Interacting	2.8	845.4	1.8	0.8	846.1	3.1	254.5	330.3	265.2
		Enriched	0	11.4	38.6	0	23.3	26.7	0	0	50

scenario LM			CRISPRi-DR predictions			MAGeCK-RRA predictions			MAGeCK-MLE predictions		
			Depleted	Non-Interacting	Enriched	Depleted	Non-Interacting	Enriched	Depleted	Non-Interacting	Enriched
	Simulated Gene Types	Depleted	42.1	7.9	0	31.2	18.8	0	50	0	0
		Non-Interacting	3.7	842.7	3.6	1.7	843.3	5	233.5	382	234.5
		Enriched	0	8.9	41.1	0	21.6	28.4	0	0	50

scenario LH			CRISPRi-DR predictions			MAGeCK-RRA predictions			MAGeCK-MLE predictions		
			Depleted	Non-Interacting	Enriched	Depleted	Non-Interacting	Enriched	Depleted	Non-Interacting	Enriched
	Simulated Gene Types	Depleted	32.9	17	0	43.7	5.7	0.6	49.3	0.7	0
		Non-Interacting	2	844.6	2.6	99.5	585.7	164.8	70.9	722	57.1
		Enriched	0	16.1	33.9	0.5	8.3	41.2	0	2.5	47.5

scenario ML			CRISPRi-DR predictions			MAGeCK-RRA predictions			MAGeCK-MLE predictions		
			Depleted	Non-Interacting	Enriched	Depleted	Non-Interacting	Enriched	Depleted	Non-Interacting	Enriched
	Simulated Gene Types	Depleted	34.4	15.6	0	30	20	0	49.9	0.1	0
		Non-Interacting	1	846.6	2.4	0.8	845.4	3.8	266.5	303.8	279.7
		Enriched	0	15.7	34.3	0	22.2	27.8	0	0	50

scenario MM			CRISPRi-DR predictions			MAGeCK-RRA predictions			MAGeCK-MLE predictions		
			Depleted	Non-Interacting	Enriched	Depleted	Non-Interacting	Enriched	Depleted	Non-Interacting	Enriched
	Simulated Gene Types	Depleted	35.3	14.7	0	32.3	17.7	0	49.9	0.1	0
		Non-Interacting	1.7	845.5	2.8	1.4	841.6	7	258.4	346.9	244.7
		Enriched	0	14.9	35.1	0	21.3	28.7	0	0.1	49.9

scenario MH			CRISPRi-DR predictions			MAGeCK-RRA predictions			MAGeCK-MLE predictions		
			Depleted	Non-Interacting	Enriched	Depleted	Non-Interacting	Enriched	Depleted	Non-Interacting	Enriched
	Simulated Gene Types	Depleted	25.9	23.9	0	43.9	5.5	0.6	49.1	0.9	0
		Non-Interacting	0.4	845.9	3	106.1	577.5	166.4	89.2	690.4	70.4
		Enriched	0	23.5	26.4	0.5	7.7	41.8	0	2.1	47.9

scenario HL			CRISPRi-DR predictions			MAGeCK-RRA predictions			MAGeCK-MLE predictions		
			Depleted	Non-Interacting	Enriched	Depleted	Non-Interacting	Enriched	Depleted	Non-Interacting	Enriched
	Simulated Gene Types	Depleted	13.3	36.7	0	33.5	16.5	0	49.6	0.3	0.1
		Non-Interacting	0.3	848.6	1.1	3.3	841.4	5.3	289	254.6	306.4
		Enriched	0	34.3	15.7	0	19.4	30.6	0	0.4	49.6

scenario HM			CRISPRi-DR predictions			MAGeCK-RRA predictions			MAGeCK-MLE predictions		
			Depleted	Non-Interacting	Enriched	Depleted	Non-Interacting	Enriched	Depleted	Non-Interacting	Enriched
	Simulated Gene Types	Depleted	13.9	36.1	0	33.5	16.2	0.3	49.9	0.1	0
		Non-Interacting	0.1	847.8	2.1	4.5	834	11.5	287.1	297.4	265.5
		Enriched	0	35	15	0	20.1	29.9	0	0.3	49.7

scenario HH			CRISPRi-DR predictions			MAGeCK-RRA predictions			MAGeCK-MLE predictions		
			Depleted	Non-Interacting	Enriched	Depleted	Non-Interacting	Enriched	Depleted	Non-Interacting	Enriched
	Simulated Gene Types	Depleted	8.9	41.1	0	43.2	6.4	0.4	48.6	1.4	0
		Non-Interacting	0	848	1.5	107.4	563.5	179.1	127.2	605.5	117.3
		Enriched	0	37.9	12.1	1	5.9	43.1	0	1.8	48.2

To clarify, the metrics are calculated as follows. For depleted genes, $Recall_D=TP_D/(TP_D+FN_D)$ and $Precision_D= TP_D/(TP_D+FP_D)$. For enriched genes, $Recall_E=TP_E/(TP_E+FN_E)$ and $Precision_E= TP_E/(TP_E+FP_E)$. For overall results, depleted and enriched genes are combined as follows: $Recall=(TP_D+TP_E)/(TP_D+FN_D+TP_E+FN_E)$ and $Precision=(TP_D+TP_E)/(TP_D+FP_D+TP_E+FP_E)$. Also, Recall=TPR, Precision=1-FPR.

		Predicted Gene Types		
		Depleted	Non-Interacting	Enriched
Simulated Gene Types	Depleted	TP_D	FN_D	
	Non-Interacting	FP_D		FP_E
	Enriched		FN_E	TP_E

Table 2 is a summary of the average recall, precision, and F1 scores for the three methods across the 9 scenarios. These scores, averaged over positive and negative interactions (excluding non-interactions), reiterate the trend observed in Table 1. As noise increases, recall rates in MAGeCK-RRA and MAGeCK-MLE increase but precision substantially decreases. Conversely, CRISPRi-DR follows a more conservative approach resulting in slightly decreasing recall rate as noise increase but maintaining consistently high precision. Across the low and medium noise scenarios, CRISPRi-DR has higher F1-scores than the other two methods where $F1\,score = 2 \times \frac{recall \times precision}{recall + precision}$ reflecting the tradeoff between recall and precision. For the higher noise scenarios, MAGeCK-RRA shows instances with greater F1-scores, than both CRISPRi-DR and MAGeCK-MLE. Note for MAGeCK-RRA, we used Fisher's method to combine the outputs from the 3 individual concentrations, as well as applied a filter of |LFC|>1 on the significant

genes. Without these additional constraints, the performance of MAGeCK-RRA is the worst of the three methods in all the noise scenarios.

Table 2. Average precision and recall values across 10 runs each for CRISPRi-DR, MAGeCK and MAGeCK-MLE for each of the 9 noise scenarios.

	CRISPRi-DR			MAGeCK-RRA			MAGeCK-MLE		
	recall	*precision*	*F1-score*	*recall*	*precision*	*F1-score*	*recall*	*precision*	*F1-score*
**LL**	0.798	0.948	0.867	0.562	0.936	0.702	0.999	0.161	0.278
**LM**	0.832	0.922	0.874	0.596	0.900	0.717	1.000	0.176	0.300
**LH**	0.669	0.939	0.781	0.858	0.243	0.379	0.968	0.433	0.598
**ML**	0.687	0.953	0.798	0.578	0.926	0.712	0.999	0.155	0.268
**MM**	0.704	0.943	0.806	0.610	0.880	0.720	0.998	0.166	0.284
**MH**	0.524	0.950	0.676	0.867	0.240	0.375	0.970	0.381	0.547
**HL**	0.290	0.964	0.446	0.641	0.885	0.743	0.993	0.143	0.250
**HM**	0.289	0.931	0.441	0.636	0.799	0.708	0.996	0.153	0.265
**HH**	0.210	0.937	0.343	0.875	0.232	0.366	0.968	0.284	0.439

The effect of noise on the results of the three methodologies can also visualized using a bar chart of true and false positives as in Fig 3 (calculated from enriched and depleted genes combined). We calculate the number of significant genes while increasing the amount of noise using either the σ_C and P_{nb} parameters (resulting in the 9 noise scenarios). In the left panel, the average number of significant genes were calculated for each method run for a specific σ_C value across the possible P_{nb} values. The errorbars seen are the 95% confidence interval of the number of significant genes. For example, the orange bar for σ_C =0.01 represents the average number of genes found significant by MAGeCK in the LL, LM, LH scenarios (where σ_C =0.01, but P_{nb} varies). The same is done in the right panel, using P_{nb}. Noise between concentration increases as σ_C is increased and noise between replicates is increases as P_{nb} is decreased.

All three methods make a comparable number of true positive calls, regardless of noise parameters. When noise increases for either parameter, the number of false positive calls for all three methods also increases. However, the number of false positives calls are higher for MAGeCK-RRA and MAGeCK-MLE than for CRISPRi-DR.

In this plot, it is clear how much more MAGeCK-RRA is affected by noise among replicates than between concentrations. The orange bar for P_{nb}=0.1 represents the results for MAGeCK-RRA in the LH, MH, HH scenarios and it shows the highest number of false positives compared to the other P_{nb} values as well as the σ_C values, consistent with the observations made in Fig 2. This is likely a result of stochastic fluctuations of counts at individual drug concentrations that are not necessarily supported at other

concentrations. This could help explain the poor performance of MAGeCK-RRA on certain datasets that are especially noise, which often generates a large number of hits; our analysis suggests that many of these hits could be false positives. CRISPRi-DR and MAGeCK-MLE are more affected by noise within concentrations than noise within replicates, since these methods rely more on increasing or decreasing trends in abundance that must be (at least somewhat) consistent across concentrations and thus is less affected by within replicate noise as MAGeCK-RRA is.

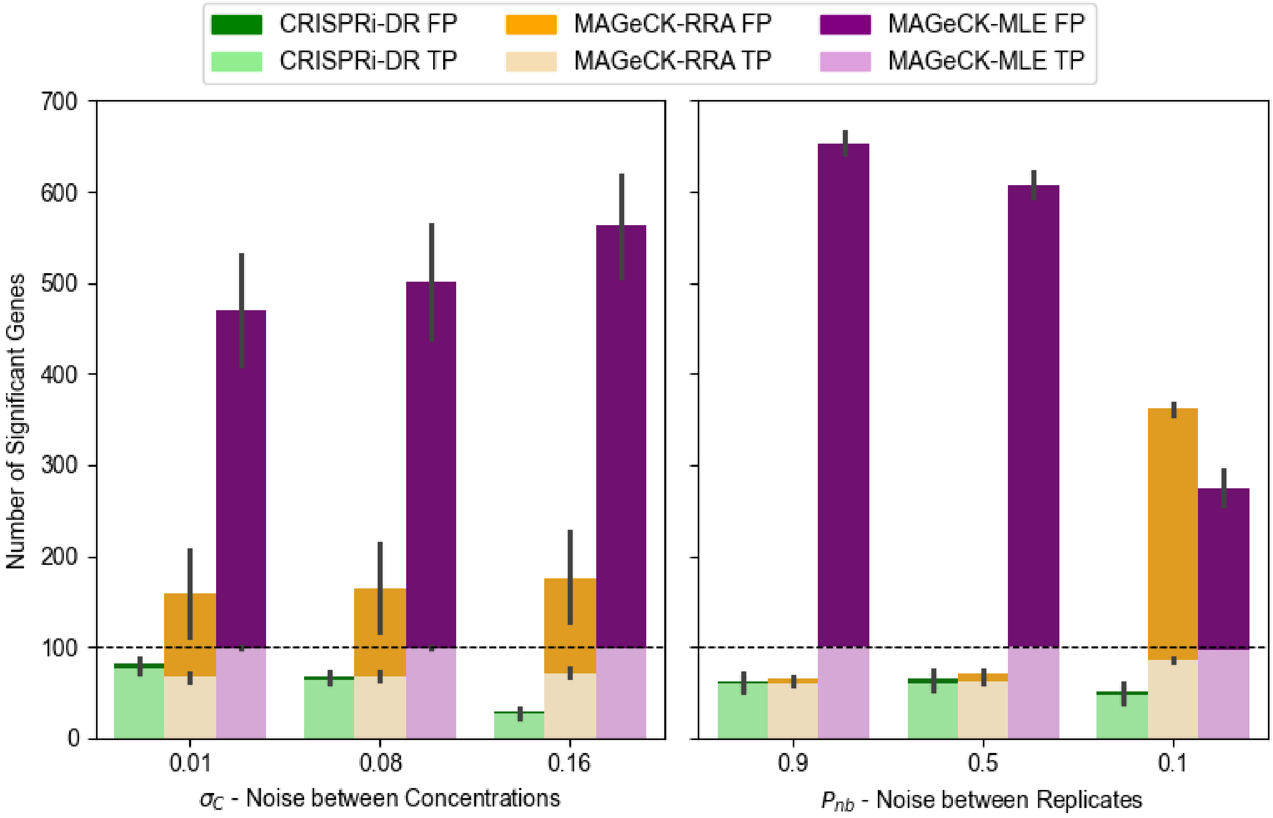

Fig 3. Bar chart of the average true positives and false positives calls for CRISPRi-DR and MAGeCK-MLE as noise parameters are adjusted to increase noise. The horizontal dashed line in both panels is the number of total simulated interacting genes (100 total). σ_C is increased to increase the noise between concentrations and P_{nb} is decreased to increase noise between replicates of concentrations. The leftmost bars of the plot are the lowest noise, and the rightmost bars are the highest noise.

Effect of K_2 (simulated concentration-dependent slopes)

The K_2 value in the simulation controls the mean of the interacting slopes in the experiment. The σ_{K2}, or the standard deviation of the simulation, is always 0.2. Fig 4 shows some of the distributions resulting from select K_2 values. As seen in Panel A with K_2=0.4, if K_2 is low, the slopes distributions of the interacting genes nearly overlap with the distribution of the slopes of the non-interacting genes. This complete overlap makes simulated genes hard to differentiate resulting in many false calls (false positives or false negatives). The other end of the K_2 range is seen in Panel C with K_2=1.2, where the slopes of non-

interacting and interacting genes have almost no overlap. This makes it easy for all methods to identify nearly all the true positives. The value we use in this simulation K_2=0.8 is seen in Panel B, that has some overlap between distributions of slopes for interacting and non-interacting genes.

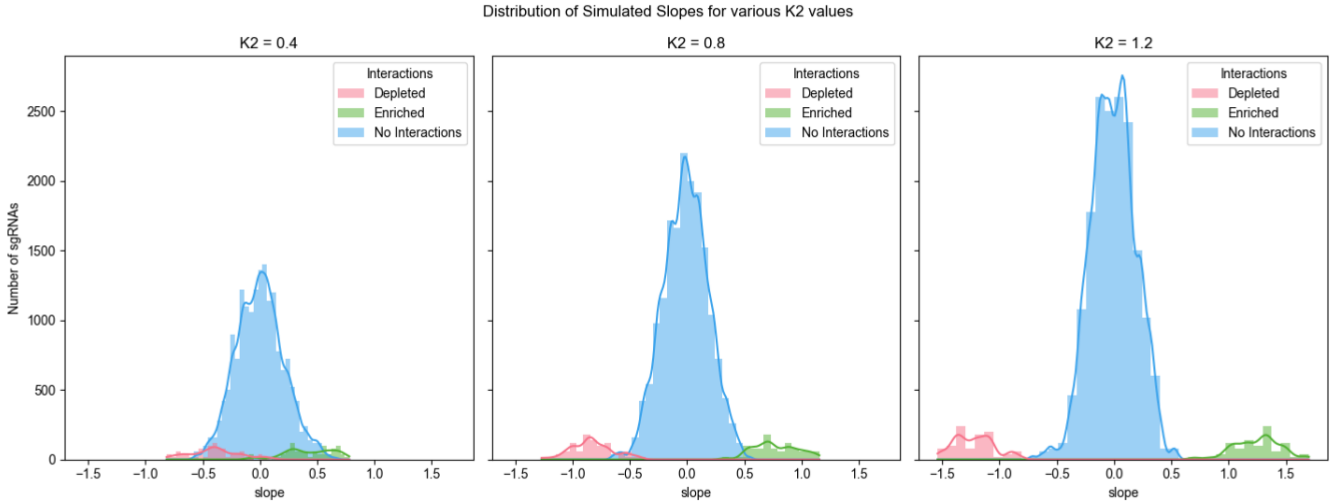

Fig 4. Distribution of slopes sampled from for simulated non-interacting and interacting genes at multiple K_2 values. The leftmost panel shows the highest amount of overlap of the distributions of simulated slopes with K_2 is low and the rightmost panel is the lowest amount of overlap of the distributions of simulated slopes with K_2 is high. The middle panel strikes a balance between these two.

Figure 5 shows the true and false positive calls made by CRISPRi-DR, MAGeCK-MLE and MAGeCK-RRA within the lowest noise scenario (LL) for a range of K_2 values, from 0.4 to 1.2. As expected, all three methods show increases in true positives and decreases in false positives as K_2 increases. Regardless of K_2 value, MAGeCK-MLE makes many more calls than CRISPRi-DR and MAGeCK-RRA and thus makes the highest number of true positive calls across the range of K_2 values but also makes a highest number of false positives calls. Comparatively, the number of false positive calls made by MAGeCK-RRA and CRISPRi-DR are low regardless of K_2 value.

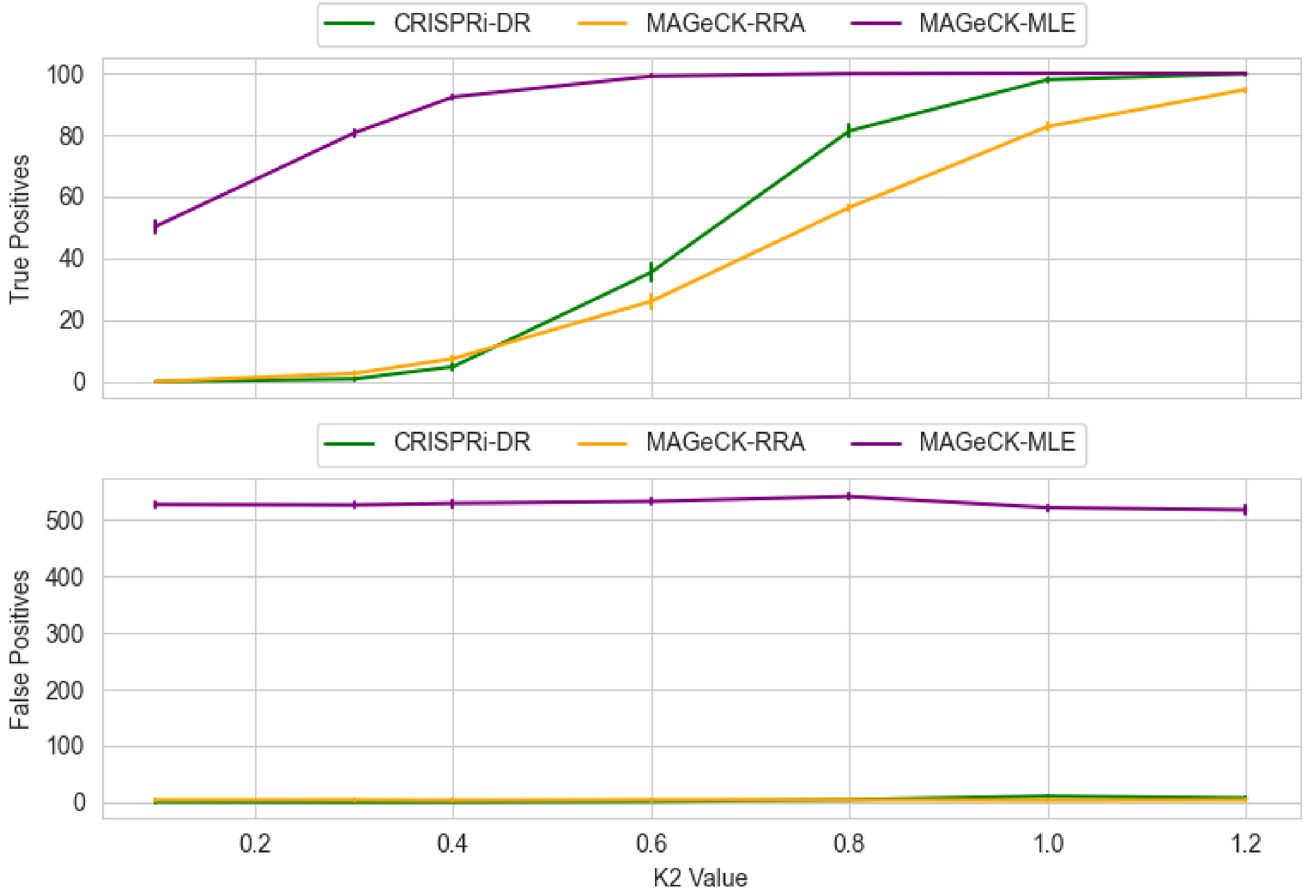

Fig 5. Calls made by CRISPRi-DR and MAGeCK-MLE at different K_2 values. CRISPRi-DR and MAGeCK-MLE were both run using the LL scenario for 3 total concentrations at a range of K_2 values. Each K_2 value was run 10 times each.

<u>Effect of Multiple Concentrations on Significant Genes detected</u>

To evaluate the benefit of profiling a CRISPRi library on multiple concentrations on the performance of CRISPRi-DR and the MAGeCK methods, we adapted the simulation above to compare their precision and recall when using one, two, or three drug concentrations. We re-ran the simulation (10 iterations per concentration amount), keeping the same concentration range (2-8 μM), and used the high-noise (HH) parameter settings (σ_C =0.16, P_{nb} =0.1). In all runs, we always kept the highest concentration (8 μM), along with the no-drug control. First, we evaluated performance using only one concentration (the highest, 8 μM), then the two highest (4 and 8 μM), then all three concentrations (the full range, 2-8 μM). For MAGeCK-RRA, each concentration was compared independently to the no-drug control, and then a single LFC value was calculated per gene as the most significant LFC across the concentrations and a single P-value was calculated per gene using the Fisher's method, which was then adjusted with Benjamini-Hochberg. For CRISPRi-DR and MAGeCK-MLE, the no-drug control was treated as the lowest concentration, and it was combined with either the highest concentration, top two, or all three drug concentrations, which were then used for doing regressions (over 2-4 effective concentration points).

As seen in Fig 6, the recall of CRISRi-DR and MAGECK-MLE (number of true positives) held constant as concentrations were added, whereas the number of true positives identified by MAGeCK-RRA increased slightly with more concentrations. Adding concentration points caused a significant increase in the number of false positives found by of MAGeCK-RRA. This is apparently due to the commitment of false positive errors, i.e. non-interacting simulated genes that are classified as significant due to high variation in barcode counts in this high noise setting. MAGeCK is susceptible to false positives when evaluating only a single concentration point, but this gets amplified as more concentrations are added, because each concentration is evaluated independently, and the hits (including false positive genes) are combined post-analysis, explaining why precision drops as concentrations are added, because errors accumulate.

In contrast, CRISPRi-DR and MAGeCK-MLE are more robust with respect to false-positive errors, because regression incorporates data from all concentrations available and looks for significant trends. This allows CRISPRi-DR to maintain higher precision, which does not decrease as additional concentration points are added.

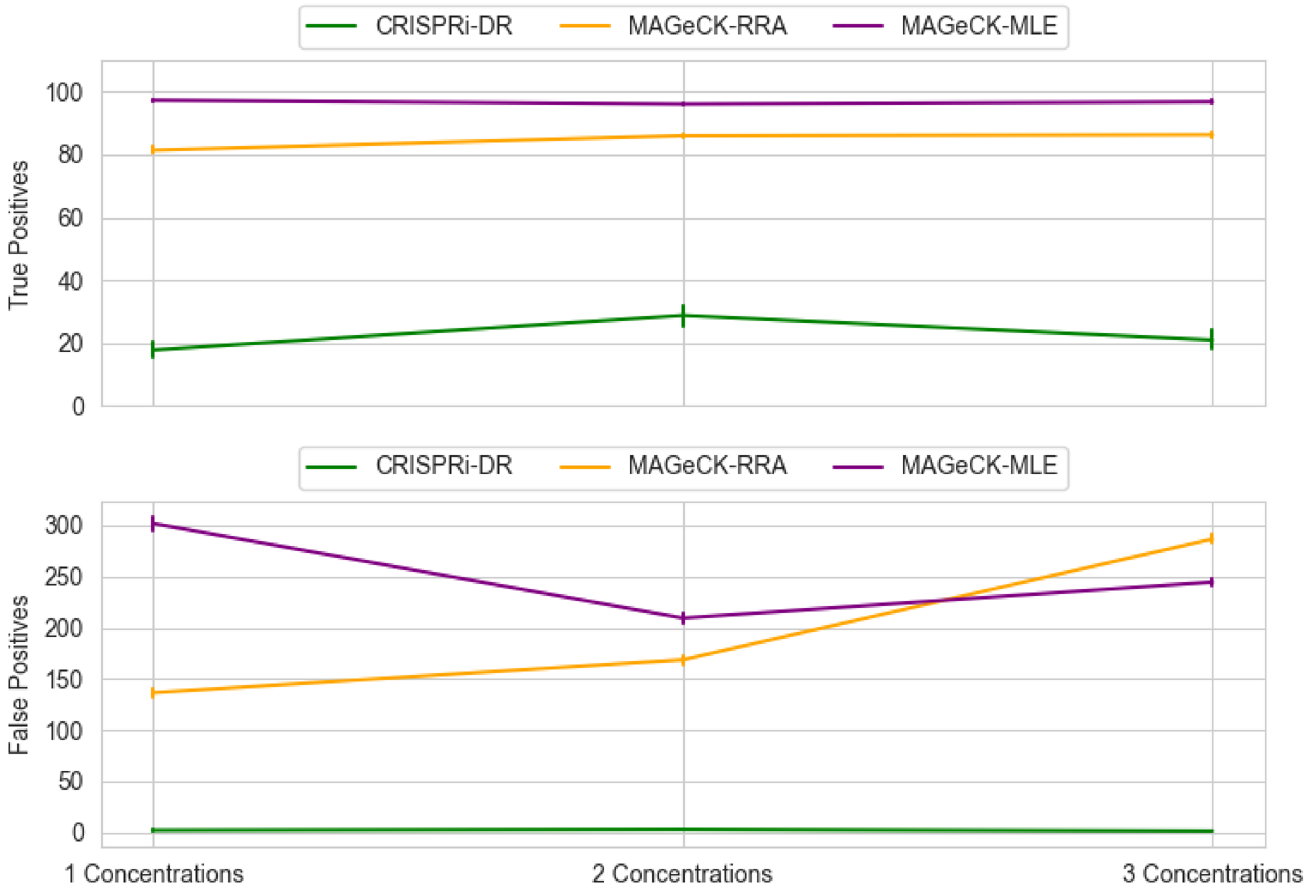

Fig 6. Changes in true and false positives with number of concentrations. CRISPRi-DR, MAGeCK-RRA, MAGeCK-MLE were all run using the HH scenario for 1, 2 and 3 total concentrations. Each concentration was run 10 times, i.e. on 10 independent simulated datasets.

<u>CRISPRi-DR can detect interacting genes where trends are not perfectly linear</u>

It is not always the case that the highest concentration should be the most informative one for detecting CGIs, as it might cause too much growth inhibition, making it difficult to assess dose-dependent behavior. Sometimes the largest effect occurs at the edge of the range, like dropping off a cliff, due to uncertainty about the optimal concentration. CRISPRi-DR can detect these kinds of trends. It only expects abundances to show an overall trend depletion or enrichment across concentrations. Fig 6 shows a few sgRNAs depicting these kinds of trends for representative sgRNAs targeting simulated interacting genes detected as significant by CRISPRi-DR. sgRNA 1 shows an example of an ideal (nearly linear) pattern of depletion. Comparatively, sgRNA 2 shows a sharp decline in abundance at concentration 1 (the lowest end of the concentration range), sgRNA 3 shows a sharp decline at concentration 2 and sgRNA 4 shows a sharp decline at concentration 3 (the highest end of the concentration range). Regardless of depletion pattern, sgRNAs 1, 2, 3 and 4 all target genes detected as significant negative interactions by the CRISPRi-DR model.

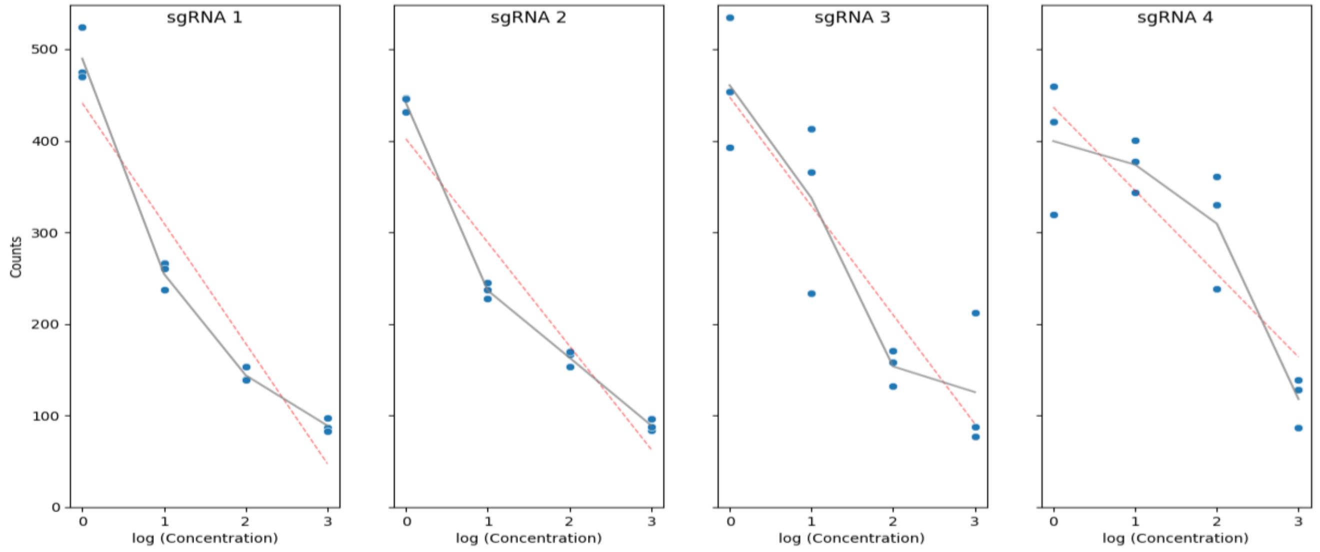

Fig 6. Trends of select sgRNAs in the various noise scenario. For each of the sgRNAs seen here, the blue dots are abundances for the three simulated replicates at each concentration. The gray line shows the change in abundance mean as concentration increases.

2. Comparison of CRISPRi-DR to other CRISPR analysis methods using CGI datasets

Algorithms Summary

The performance of CRISPRi-DR is compared to six other software packages intended for analyzing CRISPRi and CRISPRko libraries.

Below are general overviews of some existing methodologies comparable to CRISPRi-DR used to assess CRISPR screens. We describe the general math used in the analyses, as well as how we adjusted our datasets to be run using each methodology. Some methods work directly on counts, whereas others require log fold changes. The result of some of these methods is at the sgRNA level, which were combined

post hoc to obtain gene level information. Additionally, some of these methods do not take multiple concentrations into account, thus we combined outputs of analyses at different concentrations using **Fisher's method of combining P-values**:

$$p_i = \sum_{c=0}^{n} \log p_{ic} = \log(p_{i_{LOW}}) + \log(p_{i_{MED}}) + \log(p_{i_{HIGH}})$$

where n=3 for the 3 concentration levels (low, medium, high) for each gene i.

<u>DEBRA (DESeq2-based RNA-seq Analysis)</u>

R Function Call:

```
> DEBRA(counts, c("DMSO1","DMSO2", "DMSO3"), condition_names=
  c("DRUG1","DRUG2", "DRUG3"), method="DESeq2(Wald)")
```

DEBRA [3] is a methodology that uses the DESeq2 package for RNA sequencing data analysis. It processes RNA-seq count data, normalizing to account for variations in sequencing depth and biases. Then, using Negative Binomial models, DEBRA estimates gene expression variability. Through differential analysis, it identifies genes with statistically significant barcode count changes across different conditions, employing DESeq2's robust testing methods.

To use CRISPRi datasets as inputs to DEBRA, counts of the most efficient sgRNA for each gene were provided as input to DEBRA. The output of DEBRA for these datasets is log fold changes along with P-values calculated using the Ward method. Since DEBRA does not account for increasing concentration, each concentration (low, medium and high) in a given dataset was run separately, with the corresponding DMSO condition set as the control. These gene-wise P-values of the three concentration outputs were combined using the Fisher's method and then adjusted using Benjamini-Hochberg. Genes were ranked by combined P-value and marked significant if adjusted combined P-value < 0.05.

<u>CGA-LMM</u>

Command Line Prompt:

```
> Rscript ./CGA_LMM.R single_sgRNA_per_gene_counts.txt CGA_LMM_out
```

Designed for hypomorph libraries, CGA-LMM [4] assesses the concentration-dependent variation in mutant abundance (in a library) using slope coefficients derived from linear mixed models. CGA-LMM assumes one set of counts per gene and was not designed to incorporate information from multiple sgRNAs. This method uses a conservative population-based approach by identifying genes as significant only if they have slopes that are outliers when compared to the general population.

To use CRISPRi data as the input for this method, we use the most efficient sgRNA for each gene. In line with the approach of Dutta, DeJesus (4), significant genes were marked as those with adjusted P-value< 0.5 and |Zrobust| > 3.5. Genes were ranked by P-value.

CRISPhieRmix

R Function Call:

```
> CRISPhieRmix(log2fc_from_DESeq2, geneIds, negCtrl, nMesh = 100,
  PLOT = TRUE, VERBOSE = TRUE, mu=-6, pq=0.1,BIMODAL=T)
```

CRISPhieRmix [5] is a methodology created specifically for the CRISPRi variant to address guide efficiency present in CRISPRi and CRISPRa screens. The method takes log fold changes of sgRNA counts between two conditions, typically derived from DESeq2 or edgeR outputs. It fits a hierarchical mixture model on these log fold changes, based on the assumption that genes are represented by a mixture distribution of effective and ineffective guides. CRISPhieRmix then computes False Discovery Rates (FDRs) as the posterior probability that a gene is non-essential. These probabilities are aggregated over all possible mixtures to finalize the FDRs for each gene.

In analyzing our CRISPRi datasets, we utilized the *bimodal* option available in the method's implementation. Typically, this analysis resulted in one positive and one negative mode, consistent with the option's assumption that genes are a mixture of ineffective, depleting, or enriching guides. Since CRISPhieRmix does not account for increasing concentration, each concentration (low, medium, and high) in each dataset was run separately, with the corresponding DMSO condition set as the control. In the actual counts files, negative control genes were excluded, as they were causing noise in the mixture model estimations. The local FDR s from the analysis of the three concentrations were combined using the Fisher's method. Genes were ranked by were combined local FDR and marked significant if combined local FDR < 0.05.

MAGeCK (Model-based Analysis of Genome-wide CRISPR-Cas9 Knockout)

Command Line Prompt:

```
> mageck test -k drug_counts.txt -c DMSO1,DMSO2,DMSO3 -t
  CONC1,CONC2,CON3 -n Mag_out --gene-lfc-method alphamedian --norm-
  method control --control-sgrna negatives.txt
```

Li, Xu (6) designed the Robust Ranking Algorithm (RRA), one of the first algorithms for CRISPRko screens, available to researchers as MAGeCK [7]. The input to the method is raw control and experimental sgRNA counts. These counts are fitted to a Negative Binomial model to assess if counts vary significantly (similar to DESeq2). The sgRNA level P-values for each gene are the combined using a modified version of robust rank aggregation to evaluate whether a subset of them is enriched (RRA), resulting in a list of genes with False Discovery Rates (FDRs) for both positive and negative interactions.

Since MAGeCK does not account for multiple concentrations, each concentration (low, medium, and high) in a given dataset was run separately, with the corresponding DMSO condition set as the control. MAGeCK utilizes a separate set of controls be provided, from which it calculates its P-values. We provided "Negative" sgRNA controls in list form to the method for this purpose (using 1750 non-targeting sgRNAs in the Mtb CRISPRi library). For each concentration, we determined a gene's overall P-values based on the lowest FDR, whether positive or negative. We then merged the resulting gene P-values from the three concentrations using the Fisher's method and then adjusted using Benjamini-Hochberg. Genes were ranked by combined P-value and marked significant if adjusted combined P-value < 0.05.

Direct comparisons of the significant genes in the CGI libraries from Li, Poulton (1) found by CRISPRi-DR and MAGeCK-RRA can be seen in Supplemental Table S3, where significant calls made by MAGeCK-RRA have an additional constraint of |LFC|>1, to be more comparable to the publication's analysis of the data.

<u>DrugZ</u>

Command Line Prompt:

```
> drugz.py -i drug_counts.txt -o drugZ_out.txt -c DMSO1,DMSO2,DMSO3
  -x CONC1,CONC2,CONC3
```

DrugZ [8] is a method for analyzing chemical genetic interactions with drug treatments in CRISPRi, CRISPRko and CRIPSRa libraries treated with drugs. Raw sgRNA counts of the control and experimental condition are provided as input to the method. The log fold changes of the normalized counts are then calculated, along with guide level z-scores and variance estimated by empirical Bayes. The z-scores at the guide level are summed to get gene level z-scores, from which P-values can be obtained from a Normal distribution. The output of the method is similar to MAGeCK in that it provides the statistics for both the suppressive and synergistic interactions of the genes.

In our approach to analyzing CRISPRi datasets with drugZ, sgRNA counts were used directly without modification. Similar to many other methodologies, drugZ does not simultaneously accommodate multiple concentrations. Thus, each concentration (low, medium, and high) in a given dataset was run separately, with the corresponding DMSO condition set as the control. At each concentration, we determined a gene's overall P-value based on the lowest P-value, whether suppressive or synergistic. We then merged the resulting gene P-values across the three concentrations using the Fisher's method and then adjusted using Benjamini-Hochberg. Genes were ranked by were combined P-value and marked significant if adjusted combined P-value < 0.05.

<u>MAGeCK MLE</u>

Command Line Prompt:

```
> mageck mle --norm-method control -n MLE_out --genes-var 0 --
  update-efficiency --count-table subsampled_drug_counts.txt --
  threads 16 --control-sgrna negatives.txt --design-matrix
```

```
design_matrix.txt  --sgrna-efficiency squashed_sgRNA_info.txt --
sgrna-eff-name-column 1 --sgrna-eff-score-column 2
```

MAGeCK MLE [9], an extension of MAGeCK, estimates gene effects across multiple conditions (i.e. cell lines or drug treatments), while accounting for sgRNA knockout efficiency. Like MAGeCK, the input to the method is a set of raw sgRNA counts but also requires a design matrix specifying which counts come from which condition along with sgRNA efficiencies between the range of 0 and 1. Raw sgRNA counts are fitted to a Negative Binomial GLM with log link to sgRNA level counts. Maximum likelihood estimation (MLE) of fitting the guide counts across all samples is used to calculate the beta scores. The significance of these beta scores is calculated through the Wald test.

When using MAGeCK MLE with default settings, we found that a maximum of 39 sgRNAs per gene could be processed without triggering "gene too large" errors. Therefore, for genes with more than 39 sgRNAs in our CRISPRi datasets, we randomly selected 39 sgRNAs for analysis. In our design matrices, we treated increasing concentration as a time series variable. Additionally, we included sgRNA efficiencies, expressed as estimated log fold change values normalized to a 0-1 scale, where 1 represents higher sgRNA efficiency. Like MAGeCK, MAGeCK-MLE requires a list of control sgRNAs which we fulfilled with a list of “Negative” sgRNAs in each CRISPRi dataset. Genes in the MAGeCK MLE analysis results were ranked by Wald P-value and significant genes were marked as those with Wald FDR < 0.05.

Combining Results from Multiple Concentrations using Fisher’s Method for P-values

In our study, for methods that weren't designed to handle multiple concentrations simultaneously, we ran each method three times for a given CRISPRi drug dataset, once for each concentration level: low, medium, and high. We combined the significance of the genes across the three analysis results using Fisher’s method. Fig 7 illustrates the impact of this combined approach. The ROC Curves are for RIF in 1 day pre-depletion, setting target genes as the 75 conditionally essential genes (adjusted P-value < 0.5 from resampling in Transit) from a previously published TnSeq study of *M. tuberculosis H37Rv* exposed to sub-MIC concentrations of various antibiotics, including rifampicin [10]. While changes in essentiality due to transposon insertions are not technically the same as fitness defects resulting from CRISPRi depletion, there is substantial overlap between essentiality and vulnerability [2]. In the evaluation of the methods using the target genes from the TnSeq study, we only look at the 3296 non-essential genes in the *M. tuberculosis* genome, as non-essential genes are the focus of TnSeq studies.

Panel A displays ROC Curves for all runs of the tested methods, with gene rankings determined by respective P-values. For CRISPhieRmix, we used “locFDR” to rank the genes and for MAGeCK and drugZ, which provide statistics for both positive and negative interactions, we selected the minimum P-value for each gene to rank them. In this case, the high concentration runs performed the best for methods that did not account for multiple concentrations simultaneously. CRISPRi-DR performs among the best (black curve, Panel A), partly because it utilizes info from all 3 concentrations. However, one cannot just consider the highest concentration in this analysis, as sometimes the MIC of a drug can be unknown, where the highest concentration might be excessively strong, potentially leading to an overestimation of depletion effects and false positives compared to the control. In Panel B, the ROC Curves after application of the Fisher’s method closely resembled, but were not identical to, the high concentration curves in

Panel A. This indicates that lower concentrations also contribute to a gene's overall significance. Using the Fisher's method to combine significance of the three concentrations provides a more comprehensive representation of the overall significance of gene interactions across different concentration levels. Nonetheless, CRISPRi-DR still has a ROC curve that is competitive with the best of these other analysis methods (Panel B).

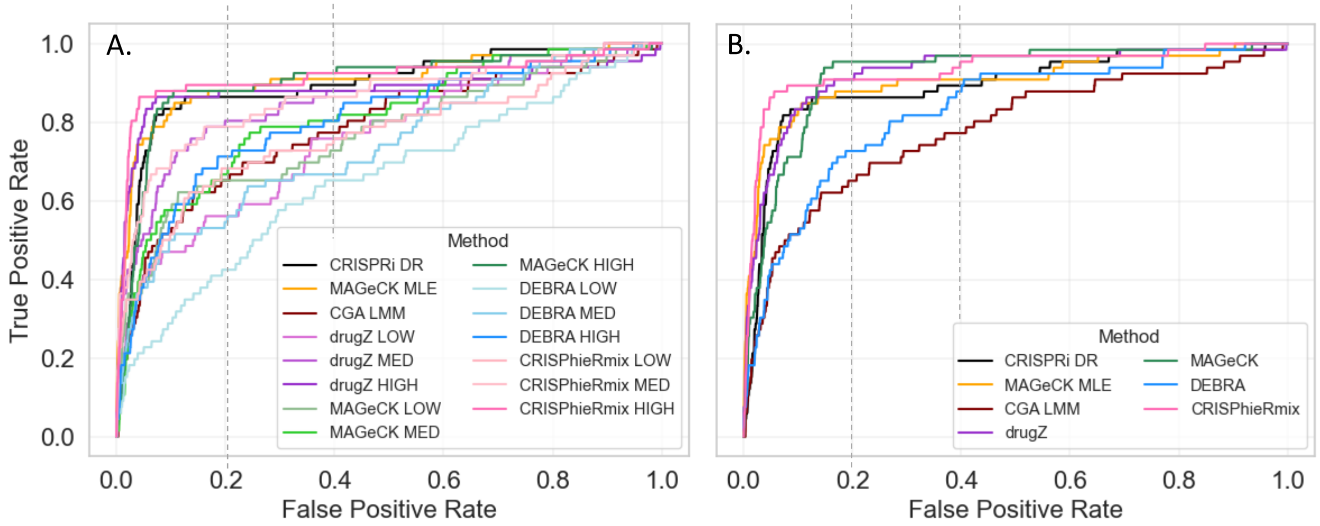

Fig 7. ROC Curves of RIF in 1 day pre-depletion using known target genes from TnSeq screens as true positives. A) ROC curves per run of a tested methodology. Rankings based on gene P-values calculated by the methodology, or with minimal post-processing. B) One ROC Curve per method based on ranking of final P-values post combining multiple concentrations for methods that do not account for them. The dotted vertical lines are at FPR=0.2 and FPR=0.4, this is the range used for the calculation of the Partial AUC.

Results

Analysis of RIF D1 CRISPRi dataset by CRISPR Methods

CRISPRi-DR performs as well as the best of the other methods in identifying the target genes by significance-based ranking (ROC curves). While the highest AUC (0.934) is achieved by CRISPhieRmix on the RIF D1 dataset, CRISRPi-DR has similar AUC of 0.894). Similarly, even in the partial AUC scores calculated for the range of FPR between 0.2 and 0.4, CRISPRi DR is among the methods with high values but is not the highest. However, the number of significant genes both by adjusted P-value < 0.05 per concentration and Fisher's combined adjusted P-value < 0.05 for the methods necessary show much higher number of false positives. The methods that find nearly all the target genes are CRISPhieRmix, MAGeCK, and MAGeCK MLE, but these methods report a very high number of false positives (thousands of genes that are putatively statistically significant). CGA-LMM detects less false positives, but it also detects only ~20 of the target genes, contributing to a low F1-score. The method comparable to the number of false positives reported by CRISPRi-DR is drugZ, both of them detect 400-600 false positives, but CRISPRi-DR detects many more target genes than drugZ. Overall, the incorporation of both sgRNA efficiency and increasing concentration in a model such as CRISPRi-DR along with significance testing

based on both the Wald test and empirical Bayes allows for rankings similar to the other CRISPR analysis techniques, but it reduces the number of false positives reported. Thus, CRISPRi-DR has the highest F1-score of the methodologies tested.

Table 3. Number of significant genes reported and AUC values of ROC Curves of RIF in 1 day pre-depletion using known target genes from TnSeq screens as true positives

Method	AUC Value	Partial AUC Value	Total Number of Significant Genes	True Positives	False Positives	F1-score
**CGA-LMM**	0.782	0.144	328	23	305	0.114
**CRISPRi-DR**	0.894	0.174	642	63	579	0.176
**CRISPhieRmix [Overall]**	0.934	0.182	3146	74	3072	0.046
**CRISPhieRmix LOW**	0.769	0.143	781	14	767	0.039
**CRISPhieRmix MED**	0.859	0.167	869	15	854	0.033
**CRISPhieRmix HIGH**	0.914	0.180	2025	41	1984	0.032
**DEBRA [Overall]**	0.831	0.159	3063	72	2991	0.046
**DEBRA LOW**	0.655	0.110	103	13	90	0.146
**DEBRA MED**	0.740	0.128	495	37	458	0.130
**DEBRA HIGH**	0.805	0.152	1960	65	1895	0.064
**MAGeCK [Overall]**	0.922	0.191	3899	75	3824	0.106
**MAGeCK LOW**	0.768	0.134	455	28	427	0.081
**MAGeCK MED**	0.812	0.155	1087	47	1040	0.080
**MAGeCK HIGH**	0.901	0.180	1634	68	1566	0.038
**MAGeCK MLE**	0.906	0.180	2833	72	2761	0.050
**drugZ [Overall]**	0.926	0.190	473	40	433	0.146
**drugZ LOW**	0.756	0.129	55	2	53	0.064
**drugZ MED**	0.851	0.166	60	4	56	0.059
**drugZ HIGH**	0.884	0.176	82	5	77	0.031

Sensitivity of CRISPR methods to noise

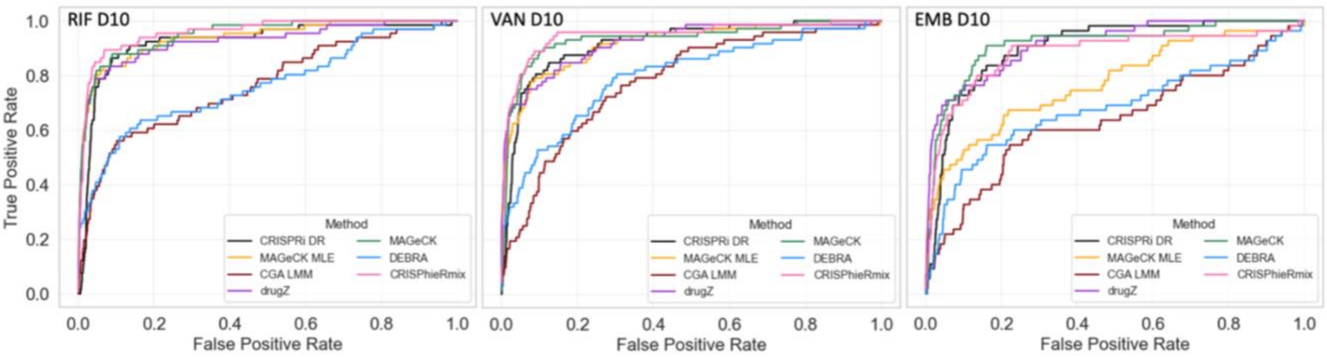

Fig 8. ROC Curves of RIF, VAN and EMB in 10 day pre-depletion using known target genes from TnSeq screens as true positives.

We assessed the performance of the methods on 10 day pre-depletion (D10) CRISPRi datasets. With greater pre-depletion days, there is expectedly more noise in the datasets. We compare the methodologies' sensitivity to higher noise through the RIF, VAN and EMB datasets with 10-day pre-depletion, using conditionally essential genes found by Xu, DeJesus (10) in their TnSeq study of antibiotic exposure as target genes. We limit the rankings of the genes by the methods to 3296 non-essential genes as non-essential genes in the focus of TnSeq studies. The ROC Curves seen in Fig 8 were generated using the ranking methods mentioned previously. Per AUCs, the performance of CRISPRi-DR remains consistently high for all three datasets. Even in high depletion datasets, CRISPRi-DR identified target genes as well as if not better than the other methodologies tested. In most cases, CRISPRi-DR found most of the target genes and had a low number of false positives, compared to the other methods. In cases such as EMB where CRISPRi-DR identified less of the target genes as significant compared to other methods, it still marked less false positive genes and thus overall had the highest F1 scores.

Table 4. Number of significant genes reported and AUC values of ROC Curves of RIF, VAN and EMB in 10 day pre-depletion using known target genes from TnSeq screens as true positives (75 genes for RIF, 89 for VAN, and 67 for EMB).

RIF D10						
Method	**AUC Value**	**Partial AUC Value**	**Total Number of Significant Genes**	**True Positives**	**False Positives**	**F1 Score**
CGA-LMM	0.762	0.132	371	25	346	0.112
CRISPRi-DR	0.923	0.187	407	62	345	0.257
CRISPhieRmix	0.959	0.192	3665	74	3591	0.040
DEBRA	0.761	0.134	2125	57	2068	0.052
MAGeCK	0.950	0.191	3596	74	3522	0.040
MAGeCK-MLE	0.938	0.187	2394	69	2325	0.056
drugZ	0.926	0.184	436	54	382	0.211
VAN D10						

Method	AUC Value	Partial AUC Value	Total Number of Significant Genes	True Positives	False Positives	F1 Score
CGA-LMM	0.781	0.144	514	27	487	0.090
CRISPRi-DR	0.919	0.185	333	59	274	0.280
CRISPhieRmix	0.948	0.191	3611	89	3522	0.048
DEBRA	0.798	0.152	2817	77	2740	0.053
MAGeCK	0.934	0.189	2849	87	2762	0.059
MAGeCK-MLE	0.915	0.183	2753	86	2667	0.061
drugZ	0.921	0.182	408	59	349	0.237
EMB D10						
Method	**AUC Value**	**Partial AUC Value**	**Total Number of Significant Genes**	**True Positives**	**False Positives**	**F1 Score**
CGA-LMM	0.636	0.115	522	17	505	0.058
CRISPRi-DR	0.901	0.184	262	35	227	0.213
CRISPhieRmix	0.880	0.180	3342	67	3275	0.039
DEBRA	0.680	0.123	2423	51	2372	0.041
MAGeCK	0.914	0.188	3913	67	3846	0.034
MAGeCK-MLE	0.773	0.138	3142	62	3080	0.039
drugZ	0.915	0.182	549	47	502	0.153

CRISPRi-DR and CGA-LMM both show higher precision than the other methods. However, CRISPRi-DR has a much higher precision than CGA-LMM, perhaps attributed to its ability to utilize both drug concentration and sgRNA efficiency in the model, whereas CGA-LMM only uses concentration. As seen in Table 5, CRISPRi-DR is the best method based on precision and F1-score for nearly all the datasets. There are quite a few methods that show high recall in the datasets (these are usually 100% recall). Precision here is calculated as TP/(FP+TP) and Recall is calculated as TP/(TP+FN). Based on AUC, MAGeCK and MAGeCK-MLE seems to have the highest values. However, Supplemental Table 2 shows that for certain datasets, such as RIF D5, the AUC values are comparably high for most methods.

Table 5. Assessment of Best CRISPR Method for the EMB, INH, LEVO, VAN and RIF CRISPRi screens in Day 1, 5 and 10 day pre-depletion based on recall, precision, AUC and F1-scrore. For the methods that require individual dosage runs, they were combined using Fisher's method across concentrations and then assessed. CRISPRi-DR is the best method by F1-Score for most of the drug-treated datasets.

Drug	Days Pre-depletion	Best Method(s) by Recall	Best Method by Precision	Best Method by AUC	Best Method by F1-Score
EMB	1	MAGeCK	drugZ	drugZ	CRISPRi DR
EMB	5	MAGeCK	drugZ	MAGeCK	drugZ
EMB	10	CRISPhieRmix, MAGeCK	CRISPRi-DR	drugZ	CRISPRi DR

INH	1	MAGeCK	CRISPRi-DR	MAGeCK	CRISPRi-DR
INH	5	MAGeCK	CRISPRi-DR	MAGeCK	CRISPRi-DR
INH	10	MAGeCK	CRISPRi-DR	MAGeCK	CRISPRi-DR
RIF	1	MAGeCK	CRISPRi-DR	drugZ	CRISPRi-DR
RIF	5	CRISPhieRmix,MAGeCK, MAGeCK MLE	drugZ	MAGeCK MLE	CRISPRi-DR
RIF	10	CRISPhieRmix, MAGeCK	CRISPRi-DR	MAGeCK	CRISPRi-DR
VAN	1	MAGeCK	CRISPRi-DR	MAGeCK MLE	CRISPRi-DR
VAN	5	CRISPhieRmix, MAGeCK	CRISPRi-DR	MAGeCK MLE	CRISPRi-DR
VAN	10	CRISPhieRmix	CRISPRi-DR	MAGeCK MLE	CRISPRi-DR
LEVO	1	DEBRA, MAGeCK	CRISPRi-DR	DEBRA	CRISPRi-DR
LEVO	5	DEBRA	CRISPRi-DR	DEBRA	CRISPRi-DR
LEVO	10	MAGeCK	CRISPRi-DR	DEBRA	CRISPRi-DR

Precision Recall Curves

Precision-Recall Curves provide an alternative visualization of performance compared to ROC Curves. We computed Precision-Recall Curves using the genes obtained from the TnSeq experiments, excluding essential genes as TnSeq focuses on non-essential genes. We used 3296 total genes in curve generation. None of the methods, including CRISPRi DR yielded ideal PR Curves. This could be attributed to the dispersion of the expected hits (~100 conditionally essential genes from the TnSeq experiments) across the ranked lists, rather than being concentrated in the top of the ranking, resulting in lower precision. (None of the methods rank the expected hits highly, as a group.) Precision, recall and F1 scores of defined significant genes for each method can be found in Supplemental Table S2

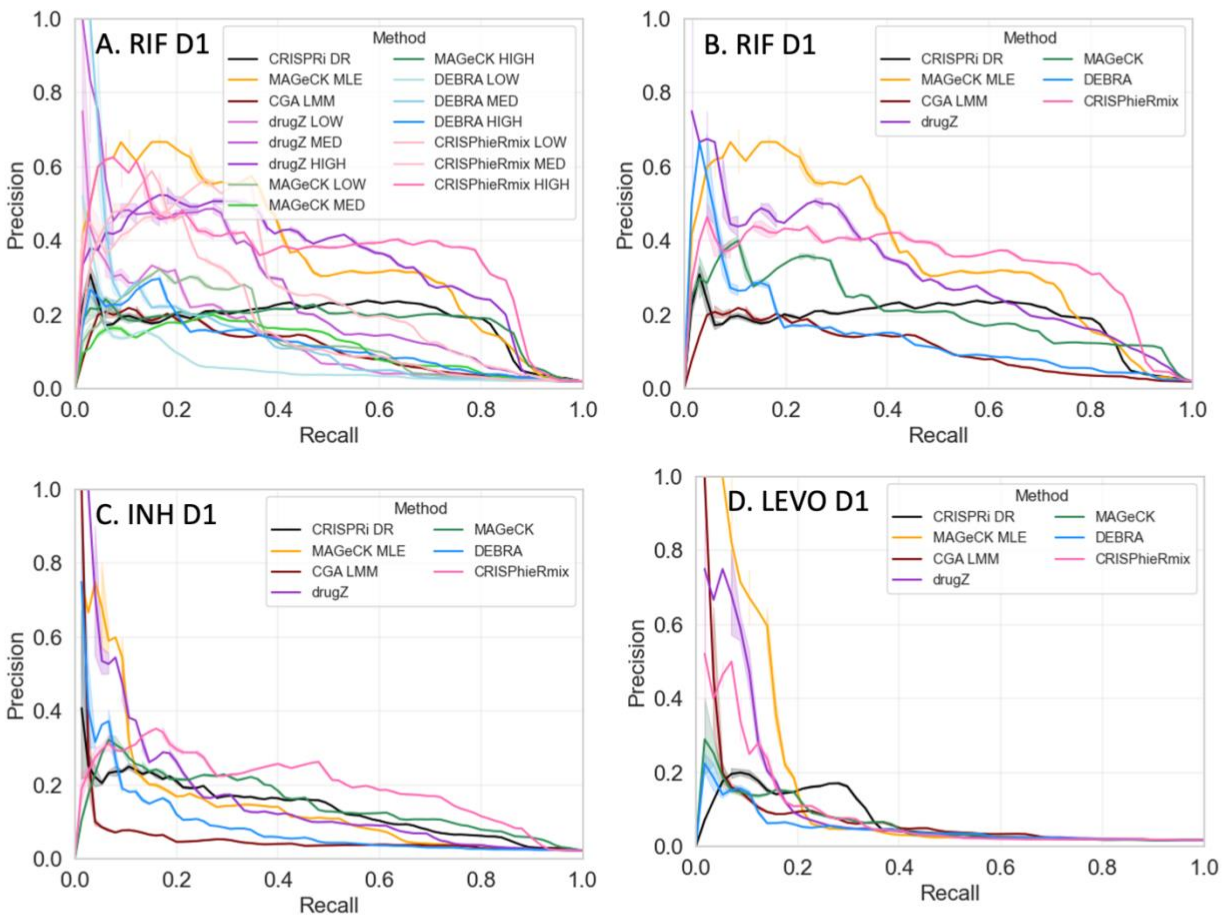

Fig 9. Precision-Recall Curves for RIF, INH and LEVO with 1 day pre-depletion. Using expected interactions derived from TnSeq studies [10] (INH and RIF) and the DNA-damage pathway (for LEVO), Precision-Recall Curves are plotted for CRISPRi-DR and 6 other CRISPR analysis methods. A) For methods that do not take concentration into account (MAGeCK, drugZ, DEBRA and CRISPhieRmix), each concentration (LOW, MED, HIGH) was analyzed independently, producing distinct ROC curves. B-D). For methods that do not take concentration into account, results of the 3 concentrations were combined using Fisher's method for combining P-values.

3. Analysis of *E. coli* CRISPRi screen using CRISPRi-DR

E. coli CRISRPi Dataset for growth on different carbon sources

Mathis, Otto and Reynolds (11) quantified growth rate changes as a function of varied gene expression levels using CRISPRi through a library of modified single guide RNAs (sgRNAs). The dataset consisted of a library of 5927 sgRNAs targeting 88 genes in *Escherichia coli MG1655*, for which they observed their effects on growth rate on media with different carbon sources. To generate diversity, they incrementally

added mutations to sgRNAs in the targeting sequence or position in gene and created showed a link between the number of mutations and their impact on growth rate.

The authors demonstrated that many gene-environment interactions not detected at maximum knockdown levels are seen at intermediate levels of expression interference. The authors quantified growth rate effects of modified sgRNAs in different carbon sources (glucose and glycerol) under turbidostat growth conditions. Compared to the conventional method of using a single (maximal-efficiency) knockdown per gene, Mathis, Otto and Reynolds (11) found 37% more interacting genes assessing the differences in the fitted parameters of a logistic fit. This fit included the quantified growth rates and the Hill coefficient.

While this is not technically a chemical-genetics (CGI) experiment, the data included multiple time points along, with sgRNAs designed to span efficiencies, satisfying requirements for our model. Thus, the abundances in this dataset can be represented through a modified version of Eq. (3) in the main text:

$$A_{ijk} = \frac{1}{1 + \left(\frac{T_{1/2}}{Time_j}\right)^{H_d} \left(\frac{K_s}{Growth\ Rate_i}\right)^{H_s}}$$

where time replaces log concentration, and growth rate replaces sgRNA efficiency. After logsigmoid transformation, the equation becomes:

$$\log\left(\frac{A_{ijk}}{1 - A_{ijk}}\right) = H_T \cdot Time_j + H_s \cdot S_i + C$$
$$C = H_s \cdot \log(K_s) - H_d \cdot T_{1/2}$$

Where the intercept folds in the inflection points $T_{1/2}$ (time results in 50% depletion) and K_s (growth rate results in 50% depletion).

Therefore two linear regressions (one for glucose and one glycerol) we fit for the dataset are :

$$\log\left(\frac{A_{ijk_{Glucose}}}{1 - A_{ijk_{Glucose}}}\right) = \beta_0 + \beta_{T_{Glucose}} \cdot Time_j + \beta_{s_{Glucose}} \cdot Growth\ Rate_i$$
$$\log\left(\frac{A_{ijk_{Glycerol}}}{1 - A_{ijk_{Glycerol}}}\right) = \beta_0 + \beta_{T_{Glycerol}} \cdot Time_j + \beta_{s_{Glycerol}} \cdot Growth\ Rate_i$$

analogous to Eq (5) in the main text for fitting sgRNA abundances for chemical genetic interaction screens.

As seen in Fig 10, the growth curves of CRISPRi knock-down mutants (depletion over time: 7 timepoints for glucose, 5 for glycerol) follows sigmoidal behavior very analogous to dose-response curves for antibiotic exposure (depletion with increasing concentration), making it a good dataset to analyze with CRISPRi-DR. In Fig 10, the changes in abundance for *fbaA* in glucose and glycerol over time can be seen. *fbaA* is a gene involved in glycolysis but not gluconeogenesis, thus being essential only for growth on

glucose. The sigmoidal curve in leftmost panel is similar to the curves seen in Fig 2 of the main text. After applying the logsigmoid transformation, both sets of curves become linear, and we can assess the time-based depletion of the gene. In glycerol, there is minimal interaction of the gene with the carbon source and thus the slope of time-based dependence post-logsigmoid transformation is slightly positive; hence, depleting *fbaA* does not produce a growth defect for growth on glycerol, in contrast to what is seen for glucose.

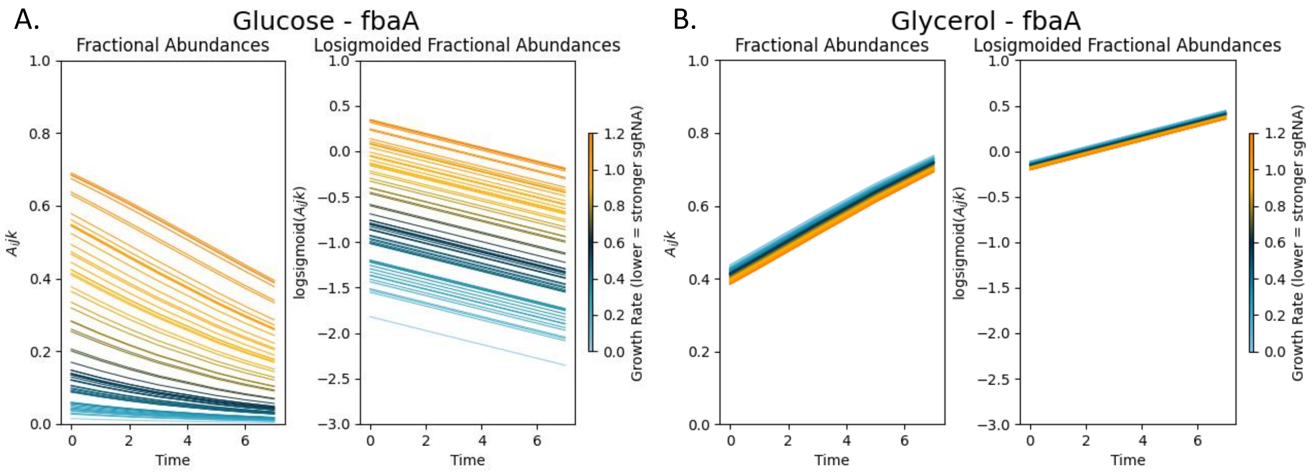

Fig 10. Time vs. Abundance Curves for *fbaA* for growth in glucose and glycerol. A) Relative abundances in *fbaA* versus time in glucose shows sigmoidal curves that can be linearized, revealing a strong depletion of *fbaA* for growth in glucose. B) Relative abundances in *fbaA* versus time in glycerol does not shows as obvious sigmoidal curves that can be linearized, revealing an almost enrichment of *fbaA* for growth in glycerol.

Predicting uninduced Abundances from SCV

The dataset generated by Mathis, Otto and Reynolds (11) quantified growth rates of the sgRNA mutants but they did not have any measurements equivalent to our uninduced abundances, i.e. abundances of the mutants in the growth mediums without ATC induction. However, the uninduced abundances could be estimated from the induced (no drug) using the SCV (standard coefficient of variation). We observed (on other datasets) that genes with greater depletion due to CRISPR interference had higher noise among their counts (abundance), which could be quantified by SCV. Fig 11 demonstrates the correlation seen of the SCV of the abundances at all concentrations is correlated with the uninduced abundances in the simulated highest noise scenario (HH). The more depleted sgRNAs have higher SCVs since lower number of total counts can increase the amount of noise. We observe similar relationships in the CRISPRi datasets as well.

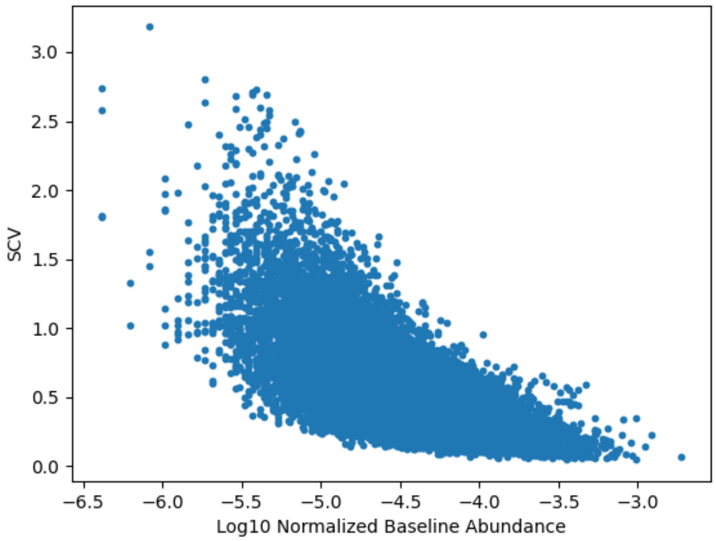

Fig 11. Correlation of SCV across concentrations vs. uninduced abundances in the simulated HH scenario. The points are linearly correlated with higher depletion resulting in higher SCV.

The calculation of the uninduced abundances using SCV of the induced counts available is:

$$SCV = \frac{stdev(Y_i)}{mean(Y_i)}$$

$$B_i = \overline{Y_{i0}} \cdot e^{\lambda \cdot SCV}$$

where B_i = baseline abundances calculated for gene i, Y_i = counts across all concentrations (Y_{i0} are counts for gene i specifically at 0 concentration) and $\lambda = 2$ for this dataset. Therefore, we used this method to estimate uninduced abundances for the *E. coli* CRISPRi data.

Results

We ran CRISPRi-DR independently for each carbon source, using the SCV method to generate uninduced baseline abundances. In both analyses, a significant number of genes exhibited notable depletion (indicating reduced fitness) or had a time-dependence coefficient q value of less than 0.05. This may be because many of the 88 genes selected for this experiment are specifically because they are essential for growth (on either carbon source).

As depicted in Fig 12A, the coefficients of time-dependence have similar distributions, they are not identical. The deviation in the distribution curves around a coefficient of -0.10 can be traced back to two genes labeled in orange in Fig 12B (*fbaA and pfkA*). In Fig 12B, we observe a strong correlation in the coefficients of time dependence of most genes; they align closely with the y=x line. These time parameter coefficients ($\beta_{T_{Glucose}}, \beta_{T_{Glycerol}}$) are analogous to the coefficients of concentration dependence is reflective of the interaction of a gene with the chemical in typical CRISPRi-DR outputs. The glycolytic

genes, highlighted fuchsia, are necessary for growth on both carbon sources. These genes are closest to the y=x line, showing similar fitness changes over time in both carbon sources, as expected. As mentioned previously, there are two notable genes (highlighted orange) *fbaA* (fructose bisphosphate aldolase) and *pfkA* (phosphofructokinase). These genes, identified in the Mathis, Otto and Reynolds (11) analysis are well-known examples of genes involved in glycolysis but not for incorporation of glycerol, as aptly have more negative coefficients in the glucose dataset analysis than the glycerol.

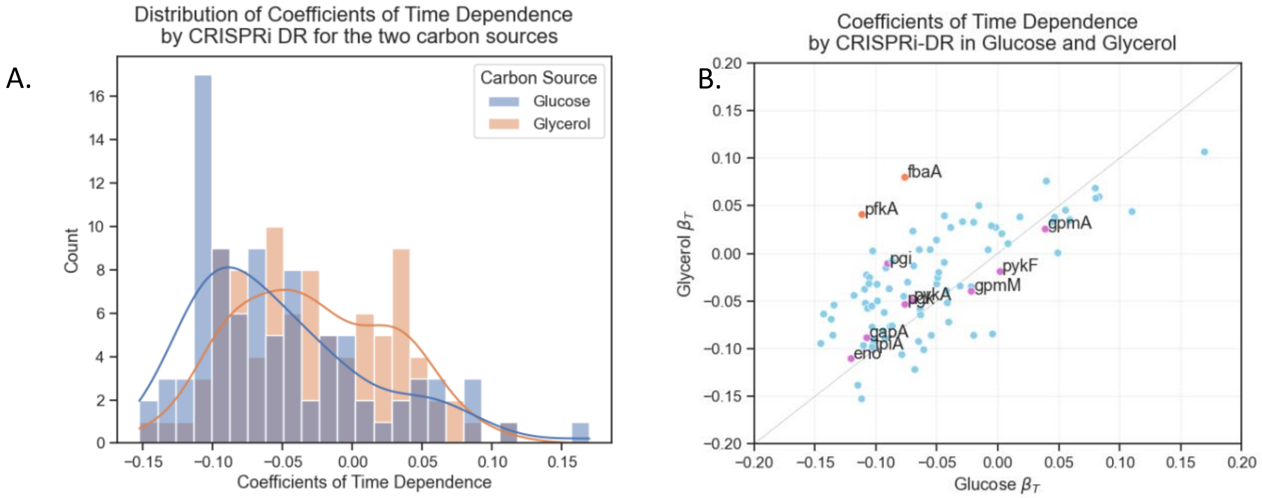

Fig 12. Coefficients of Time-Dependence in CRISPRi-DR Analyses of Glucose and Glycerol Data. A) Distribution of the Coefficients of Time Dependence. B) Correlation plot of the coefficients of the genes. The solid diagonal line is y=x. The fuschia labeled points closest to the line are genes involved in both gluconeogenesis and glycolysis. The points farther away from this line, the orange labeled points (*pfkA* and *fbaA),* are genes involved in glycolysis but not gluconeogenesis and they have more negative coefficients in glucose than in glycerol.

The analysis of this dataset demonstrates that the CRISPRi-DR method can be applied to other datasets, including those not explicitly designed for chemical-genetics. The modified Dose-Response model nicely incorporates the simultaneous effects of time and the variable efficiencies of sgRNAs of varying effiency on mutant abundance.

4. Minimum Number of sgRNAs per gene needed for CRISPRi-DR

Creating a library of sgRNAs can be expensive and time-consuming. A user may want to know how many sgRNAs per gene are necessary to span a range of predicted efficiencies and reflect genuine interactions with a given treatment. Based on our investigation below, we recommend at least 5 sgRNAs per gene.

We subsampled our existing library of about 96,000 total sgRNAs such that each gene has a maximum of 2, 4, 6, 8, 10, and so on sgRNAs per gene. We re-rank the genes after running these subsampled libraries through the CRISPRi-DR model. ROC Curves with target genes obtained from Xu, DeJesus (10) reveals

that 2 sgRNAs per gene is not enough to capture expected interactions, but at least 5 sgRNAs spanning a range of predicted efficiencies is sufficient.

Rankings of Select Genes with Reduced Sets of sgRNAs

In a library treated with isoniazid at 1 day pre-depletion, we sampled all the genes to have a maximum of 2-20 sgRNAs, incrementing at intervals of 2, and ran the sampled libraries through the CRISPRi-DR model. We repeated this sampling 10x each at every increment.

In Panel A of Fig 13, we see *inhA* (enoyl-ACP reductase, in mycolic acid pathway) is an essential gene that is the target of INH [12], and *nadA*, an enriched gene in this dataset (with all sgRNAs). The figure depicts changes in ranking of these two genes as the number of sampled sgRNAs is increased. Depletion ranking in this context is defined as genes in the order of increasing concentration dependence slope and enrichment ranking is genes in the order of decreasing concentration dependence slope. With all the sgRNAs, the depletion ranking of *inhA* is #12 and the enrichment ranking of *nadA* is #21. The shaded region surrounding the line is the standard deviation of the rankings across the 10 iterations performed at a particular sampling level. At the left end of the plot, with a low number of sgRNAs sampled per gene, the standard deviations are high, at 30.2 for *inhA* and 134.6 for *nadA*. These standard deviations reduce sustainably and rankings for both genes start to converge to their true rankings (based on all sgRNAs) at around the 5 sgRNA sampling level.

We observed a similar phenomenon in Panel B of Fig 13, with *rpoC* and *eccD3,* a few genes that interact with rifampicin with 1 day pre-depletion. The variation of the rankings of these genes is possibly more variable than those in the isoniazid library depicted in Panel A, with a standard deviation of 283.8 for *eccD3* and 202.6 for *rpoC* at the 2 sgRNA sampling level. However, the rankings of these genes also converge at the 5 sgRNA subsampling level.

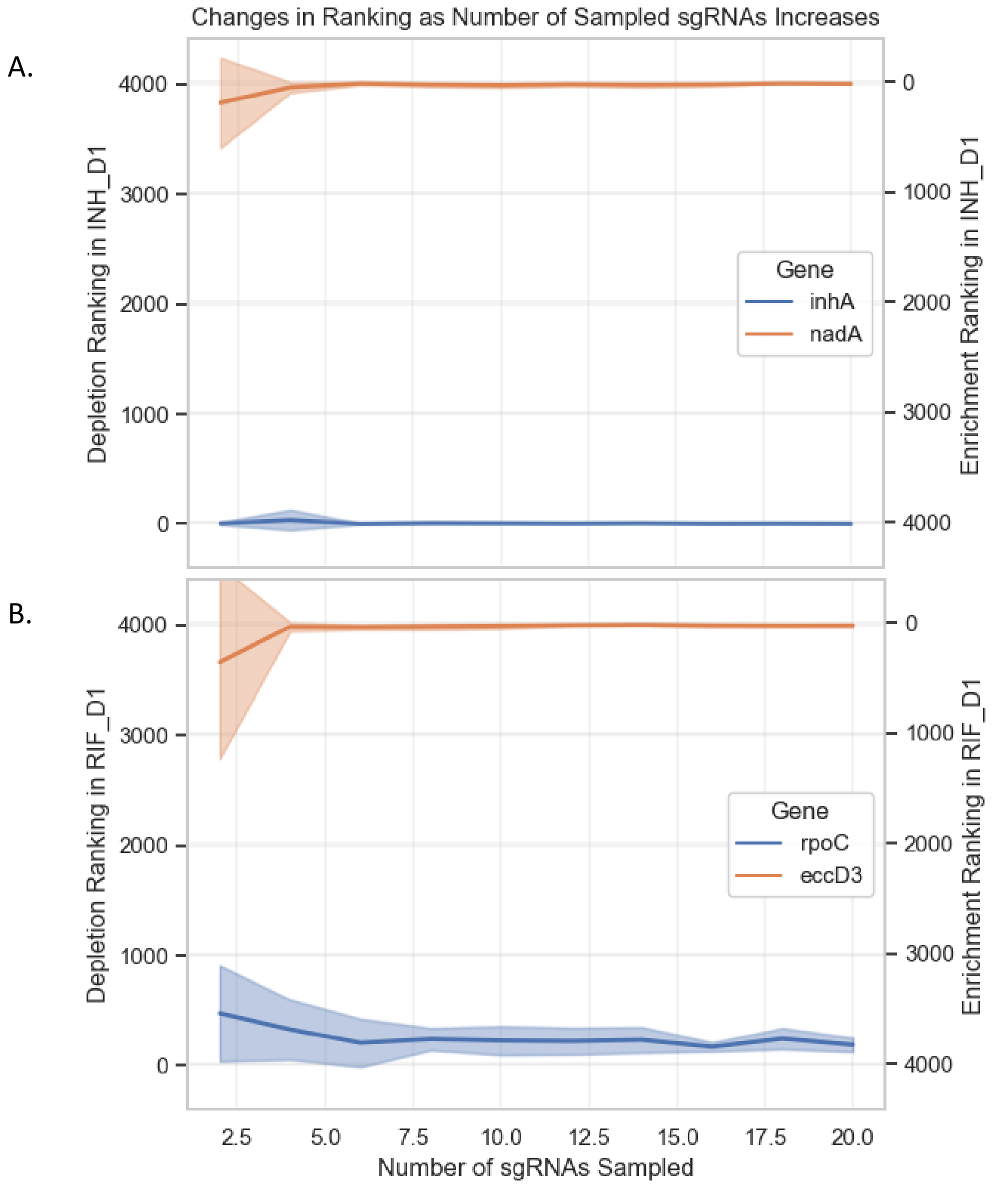

Fig 13. The rankings for select genes based on maximum number of sgRNAs sampled per gene. (A) select genes from INH 1 day pre-depletion. *inhA* is significantly depleted and *nadA* is significantly enriched in the presence of isoniazid (B) Select genes from RIF 1 day pre-depletion. *rpoC* is highly depleted and *eccD3* is highly enriched in the presence of rifampicin. Each sampled sgRNA library is run through the CRISPRi-DR model 10 times. The shaded regions represent the standard deviations of rankings over 10 iterations at every sgRNA sampling value. In both panels, gene rankings converge at about 5 sgRNAs.

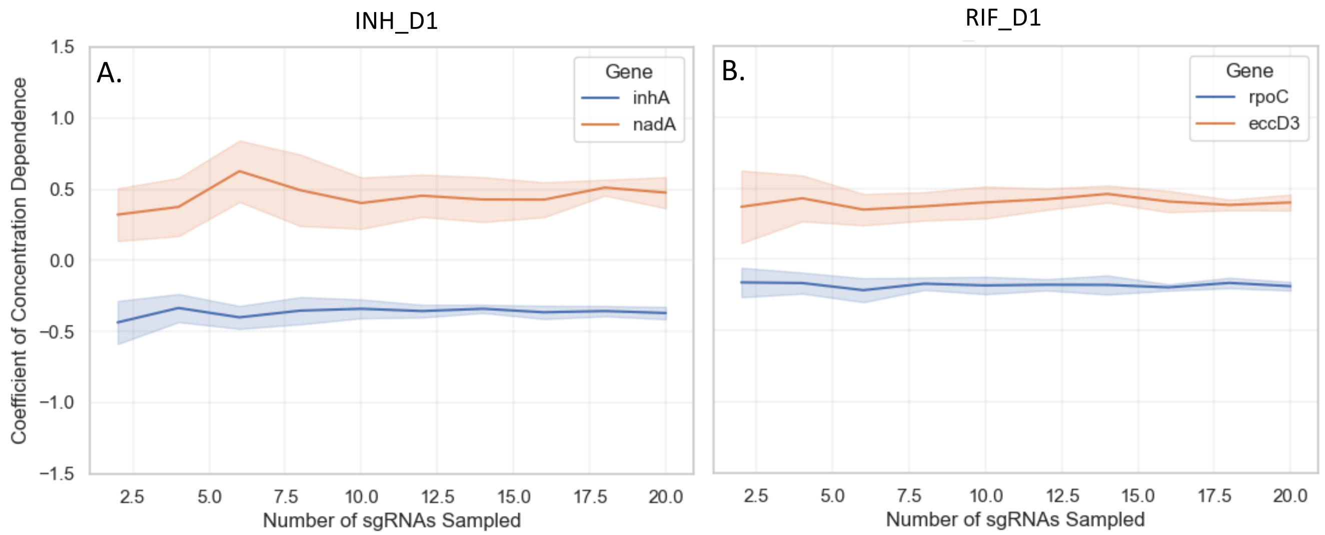

Fig 14. Coefficient of concentration dependence select genes based on maximum number of sgRNAs sampled per gene. (A) select genes from INH 1 day pre-depletion. (B) Select genes from RIF 1 day pre-depletion Each sampled sgRNA library is run through the CRISPRi-DR model 10 times and the slopes of concentration dependence extracted for the select genes.

Too few sgRNAs could reduce the stability of the regressions and lead to less accurate (or more variable) estimates of slope coefficients. As seen in Fig 15, the r^2 values of the select genes show a high standard deviation when less than 5 sgRNAs are sampled (which means the slope coefficients indicating concentration-dependence vary depending on which sgRNAs are selected see Fig 14), which in turn impedes the model's ability to detect significant interactions.

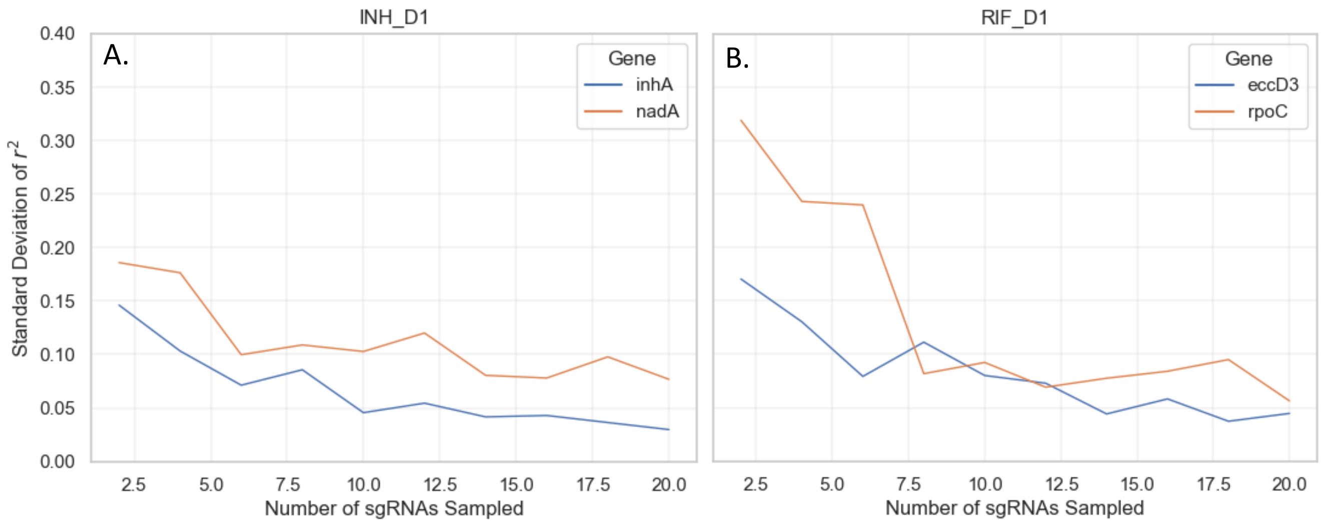

Fig 15. Standard Deviations of r^2 for fitting of select genes based on maximum number of sgRNAs sampled per gene. (A) select genes from INH 1 day pre-depletion. (B) Select genes from RIF 1 day pre-depletion Each sampled sgRNA library is run through the CRISPRi-DR model 10 times and the r^2 values extracted for the select genes. The standard deviations of these r^2 values are obtained and for each sampling level and plotted here. In both panels, standard deviation starts to converge at about 5 sgRNAs.

ROC Curves of Subsampled Libraries

Using target genes from Xu, DeJesus (10) for INH and RIF, ROC Curves were generated for each of the sampled sgRNA libraries. As Fig 16 shows, in both RIF and INH, the lowest performing library is that where 2 sgRNAs are sampled for all genes in INH and RIF screens. After that (6 sgRNAs and upward), performance remains consistently high. Combined with the ranking changes observed in Fig 13 and the changes in standard deviation of r^2 values observed in Fig 15, this suggests that each target gene should be represented by a minimum of 5 sgRNAs in designing a CRISPRi library.

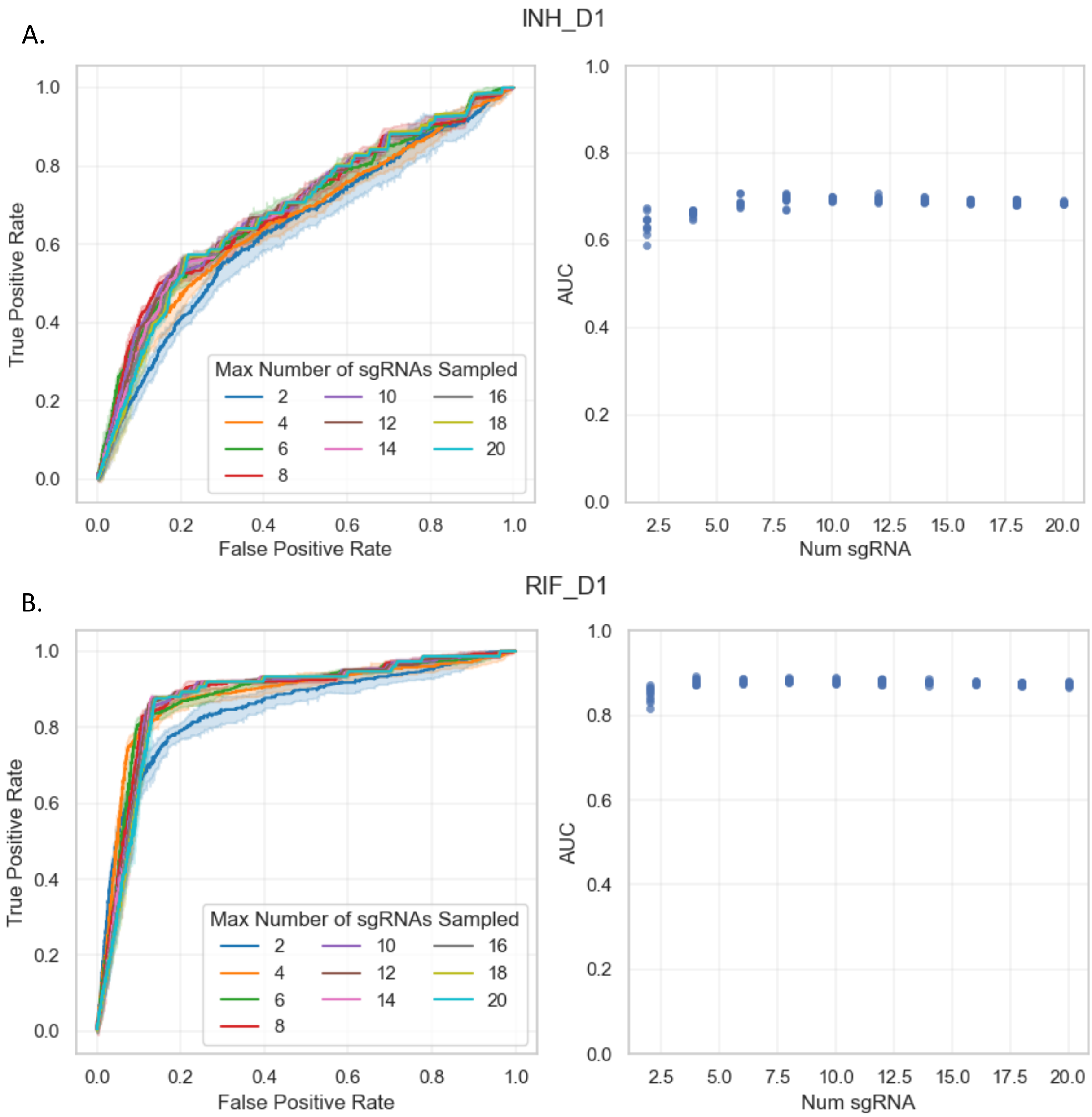

Fig 16. ROC Curves for each of the maximum number of sgRNAs sampled. (A) ROC Curves for the different number of maximum sgRNAs sampled from the INH 1 day pre-depletion, along with the AUC values. (B) ROC Curves for the different number of maximum sgRNAs sampled from the RIF 1 day pre-

depletion, along with the AUC values. The shaded regions surrounding the lines is the standard deviation of the true positive rate at each false positive rate. In both Panels, the worst performing sgRNA sampling amount is 2, with the lowest AUC across the 10 runs. Starting at 4 sgRNAs, the performance of the library remains constant.

Thus, we conclude that at least 5 sgRNAs (of diverse efficiency) should be included for each gene when designing a CRISPRi library, to ensure adequate regression fits with CRISPRi-DR.

References

1. Li S, Poulton NC, Chang JS, Azadian ZA, DeJesus MA, Ruecker N, et al. CRISPRi chemical genetics and comparative genomics identify genes mediating drug potency in Mycobacterium tuberculosis. Nat Microbiol. 2022;7(6):766-79. Epub 20220530. doi: 10.1038/s41564-022-01130-y. PubMed PMID: 35637331; PubMed Central PMCID: PMCPMC9159947.
2. Bosch B, DeJesus MA, Poulton NC, Zhang W, Engelhart CA, Zaveri A, et al. Genome-wide gene expression tuning reveals diverse vulnerabilities of M. tuberculosis. Cell. 2021;184(17):4579-92 e24. Epub 20210722. doi: 10.1016/j.cell.2021.06.033. PubMed PMID: 34297925; PubMed Central PMCID: PMCPMC8382161.
3. Akimov Y, Bulanova D, Timonen S, Wennerberg K, Aittokallio T. Improved detection of differentially represented DNA barcodes for high-throughput clonal phenomics. Mol Syst Biol. 2020;16(3):e9195. doi: 10.15252/msb.20199195. PubMed PMID: 32187448; PubMed Central PMCID: PMCPMC7080434.
4. Dutta E, DeJesus MA, Ruecker N, Zaveri A, Koh EI, Sassetti CM, et al. An improved statistical method to identify chemical-genetic interactions by exploiting concentration-dependence. PLoS One. 2021;16(10):e0257911. Epub 20211001. doi: 10.1371/journal.pone.0257911. PubMed PMID: 34597304; PubMed Central PMCID: PMCPMC8486102.
5. Daley TP, Lin Z, Lin X, Liu Y, Wong WH, Qi LS. CRISPhieRmix: a hierarchical mixture model for CRISPR pooled screens. Genome Biol. 2018;19(1):159. Epub 20181008. doi: 10.1186/s13059-018-1538-6. PubMed PMID: 30296940; PubMed Central PMCID: PMCPMC6176515.
6. Li W, Xu H, Xiao T, Cong L, Love MI, Zhang F, et al. MAGeCK enables robust identification of essential genes from genome-scale CRISPR/Cas9 knockout screens. Genome Biol. 2014;15(12):554. doi: 10.1186/s13059-014-0554-4. PubMed PMID: 25476604; PubMed Central PMCID: PMCPMC4290824.
7. Bodapati S, Daley TP, Lin X, Zou J, Qi LS. A benchmark of algorithms for the analysis of pooled CRISPR screens. Genome Biol. 2020;21(1):62. Epub 20200309. doi: 10.1186/s13059-020-01972-x. PubMed PMID: 32151271; PubMed Central PMCID: PMCPMC7063732.
8. Colic M, Wang G, Zimmermann M, Mascall K, McLaughlin M, Bertolet L, et al. Identifying chemogenetic interactions from CRISPR screens with drugZ. Genome Med. 2019;11(1):52. Epub 20190822. doi: 10.1186/s13073-019-0665-3. PubMed PMID: 31439014; PubMed Central PMCID: PMCPMC6706933.
9. Li W, Koster J, Xu H, Chen CH, Xiao T, Liu JS, et al. Quality control, modeling, and visualization of CRISPR screens with MAGeCK-VISPR. Genome Biol. 2015;16:281. Epub 20151216. doi: 10.1186/s13059-015-0843-6. PubMed PMID: 26673418; PubMed Central PMCID: PMCPMC4699372.
10. Xu W, DeJesus MA, Rucker N, Engelhart CA, Wright MG, Healy C, et al. Chemical Genetic Interaction Profiling Reveals Determinants of Intrinsic Antibiotic Resistance in Mycobacterium tuberculosis. Antimicrob Agents Chemother. 2017;61(12). Epub 20171122. doi: 10.1128/AAC.01334-17. PubMed PMID: 28893793; PubMed Central PMCID: PMCPMC5700314.
11. Mathis AD, Otto RM, Reynolds KA. A simplified strategy for titrating gene expression reveals new relationships between genotype, environment, and bacterial growth. Nucleic Acids Res.

2021;49(1):e6. doi: 10.1093/nar/gkaa1073. PubMed PMID: 33221881; PubMed Central PMCID: PMCPMC7797047.
12. Vilcheze C, Jacobs WR, Jr. The mechanism of isoniazid killing: clarity through the scope of genetics. Annu Rev Microbiol. 2007;61:35-50. doi: 10.1146/annurev.micro.61.111606.122346. PubMed PMID: 18035606.
